# **RNA-seq analysis of differential gene expression in liver from lactating dairy cows divergent in negative energy balance**

**DOI:** 10.1186/1471-2164-13-193

**Published:** 2012-05-20

**Authors:** Matthew McCabe, Sinéad Waters, Dermot Morris, David Kenny, David Lynn, Chris Creevey

**Affiliations:** 1 Animal and Bioscience Research Department, Animal and Grassland Research and Innovation Centre, Teagasc, Grange, Dunsany, Co. Meath, Ireland; 2 Animal and Bioscience Research Department, Animal and Grassland Research and Innovation Centre, Teagasc, Mellows Campus, Athenry, Co. Galway, Ireland

**Keywords:** RNA-seq, Negative energy balance, Bovine, Liver

## Abstract

**Background:**

The liver is central to most economically important metabolic processes in cattle. However, the changes in expression of genes that drive these processes remain incompletely characterised. RNA-seq is the new gold standard for whole transcriptome analysis but so far there are no reports of its application to analysis of differential gene expression in cattle liver. We used RNA-seq to study differences in expression profiles of hepatic genes and their associated pathways in individual cattle in either mild negative energy balance (MNEB) or severe negative energy balance (SNEB). NEB is an imbalance between energy intake and energy requirements for lactation and body maintenance. This aberrant metabolic state affects high-yielding dairy cows after calving and is of considerable economic importance because of its negative impact on fertility and health in dairy herds. Analysis of changes in hepatic gene expression in SNEB animals will increase our understanding of NEB and contribute to the development of strategies to circumvent it.

**Results:**

RNA-seq analysis was carried out on total RNA from liver from early post partum Holstein Friesian cows in MNEB (n = 5) and SNEB (n = 6). 12,833 genes were deemed to be expressed (>4 reads per gene per animal), 413 of which were shown to be statistically significantly differentially expressed (SDE) at a false discovery rate (FDR) of 0.1% and 200 of which were SDE (FDR of 0.1%) with a ≥2-fold change between MNEB and SNEB animals. GOseq/KEGG pathway analysis showed that SDE genes with ≥2- fold change were associated (*P* <0.05) with 9 KEGG pathways. Seven of these pathways were related to fatty acid metabolism and unexpectedly included ‘Steroid hormone biosynthesis’, a process which mainly occurs in the reproductive organs rather than the liver.

**Conclusions:**

RNA-seq analysis showed that the major changes at the level of transcription in the liver of SNEB cows were related to fat metabolism. 'Steroid hormone biosynthesis', a process that normally occurs in reproductive tissue, was significantly associated with changes in gene expression in the liver of SNEB cows. Changes in gene expression were found in this pathway that have not been previously been identified in SNEB cows.

## Background

Selective breeding for high milk yield in dairy cows has led to breeds in which the nutritional demands of the very high lactation rates following calving are in excess of that which the animal can metabolise from ingested feed [1]. This aberrant physiological state is known as negative energy balance (NEB) and is of particular economic importance in the dairy industry because of its negative impact on health and fertility [1].

The principal physiological response of animals to NEB is to try and maintain homeostasis by mobilising body fat (and protein) reserves. This results in the release of non-esterified fatty acids (NEFAs) from adipose. NEFAs are transported primarily to the liver where they are either fully oxidized to CO_2_, or converted to ketone bodies such as beta-hydroxybutyrate (BHB), or esterified into triacylglycerides (TAG) either for delivery into blood as very low density lipoproteins proteins (VLDL) or storage as cytosolic lipid droplets [2]. As with all ruminants, dairy cattle have very low rates of VLDL synthesis and secretion [3] and are therefore susceptible to accumulation of high levels of TAG in the liver cells (referred to as fatty liver or lipidosis) and high concentrations of ketone bodies in the blood (ketosis) [4] both of which are potentially detrimental to the health of the animals [5]. Although there are strategies to prevent and treat NEB, such as management of diet and inclusion of dietary supplements [6], [7] NEB and ketosis continue to have a significant negative economic impact in dairy cattle. This has led to an effort to understand NEB at the level of genes and their expression [8].

As with the majority of metabolic states, changes in gene expression in the liver are central to NEB. There have been several studies describing such changes at the level of transcription in the liver of NEB cows which have employed qPCR [9] and bovine microarrays [10,11]. However, microarray analyses rely on existing genome annotations and require design of a specialised array of probes based on information obtained from other methods such as sequencing in order to detect more complex regulations in gene expression such as alternative splicing [12]. Furthermore, the insensitivity of microarray platforms in detecting differential expression of certain metabolically important genes has been previously highlighted by our own group who found that qPCR was able to detect changes in hepatic gene expression SNEB cows which microarray analysis failed to detect [11]. This emphasises the need for a more sensitive technique for whole transcriptome analysis.

RNA-seq is a relatively new technique that can be used to analyse changes in gene expression across the entire transcriptome [13,14], and is now being applied to a rapidly increasing number of organisms [12]. This technology has distinct advantages over microarrays including the sensitive detection of all expressed genes without the need to generate an array of probes based on known sequence, virtually no background noise and a much higher dynamic range. It has also led to the discovery of previously unidentified transcripts such as the recent discovery of enhancer RNA, a novel RNA species [15] and as the transcriptome is being sequenced, alternative splicing [16], single nucleotide polymorphisms (SNPs) [17], transcriptional fusions and chimeric transcripts [18] can be detected without the need to design an array of probes.

Unlike microarrays, the bioinformatic tools for the analysis RNA-seq data are still at the early stages of development [19,20]. Partly for this reason, there are few reports to date on the use of RNA-seq for identification of significantly differentially expressed (SDE) genes between physiologically different groups of animals and, so far, no such reports for cattle. The use of RNA-seq to identify differential gene expression in two pools of cattle embryos that were divergent for viability was recently reported [12]. However, as RNA from two pools of 20 embryos was analysed rather than individual embryos, the authors were unable to apply statistical analysis to their data to determine statistical significance of the differential gene expression.

Here we describe the use of DEseq [21] and GOseq [22] to identify SDE genes and associated over-represented biological KEGG pathways across the whole liver transcriptome of individual cattle. For this we compared RNA samples from liver of mild and severe NEB (MNEB and SNEB) cows. The objective of our study was to use RNA-seq to identify novel genes and their associated biological pathways which are important in SNEB in cattle.

## Results and discussion

### Library preparation and sequencing of polyA mRNA-seq libraries

Twelve polyA RNA-seq single read libraries that had been prepared from total RNA extracted from liver from 6 MNEB animals (libraries 1–6) and 6 SNEB animals (libraries 7–12) were run as 36 bp single reads on 21 lanes randomly distributed across 3 flowcells (Table 1). Several libraries were run on one or more lanes at the same or different concentrations to determine the effect of library concentration on cluster generation and to assess the consistency of sequence between different lanes and flow cells. For the 21 lanes, the average number of raw reads per lane was 13.66 million. Reads were aligned to the BCM4 genome assembly [23], using the ultrafast short read aligner, Bowtie (version 0.12.3) [24]. Following removal of reads that either failed to align to BCM4 or aligned to more than one position in the genome, and the removal of all but one of the reads that mapped to exactly the same position on the genome (i.e. putative PCR duplicates), an average of 3.8 million reads per lane were retained for read abundance calculation. An overview of these data is given in Additional file 1 and they have been deposited in NCBI's Gene Expression Omnibus and are accessible through GEO Series accession number GSE37544 (http://www.ncbi.nlm.nih.gov/geo/query/acc.cgi?acc=GSE37544).

**Table 1:** Summary of RNA-seq libraries and flow cells

**Lane**	**Flow cell A**	**Lane**	**Flow cell B**	**Lane**	**Flow cell C**
1A	library 1 (4.5 pM)	1B	library 12 (5.5 pM)	1 C	library 2 (5.5 pM)
2A	library1 (5.0 pM)	2B	library 6 (5.5 pM)	2 C	library 3 (5.5 pM)
3A	library 5 (5.5 pM)	3B	library 2 (5.5 pM)	3 C	library 6 (5.5 pM)
4A	library5 (6.0 pM)	4B	library 7 (5.5 pM)	4 C	library 7 (5.5 pM)
5A	library 10 (4.0 pM)	5B	library 9 (5.5 pM)	5 C	PhiX
6A	PhiX	6B	PhiX	6 C	library 8 (5.5 pM)
7A	library 10 (5.5 pM)	7B	library 11 (5.5 pM)	7 C	library 11 (5.5 pM)
8A	library 10 (6.5 pM)	8B	library 4 (5.5 pM)	8 C	library 12 (5.5 pM)

Libraries 1–6 = mild negative energy balance animals. Libraries 7–12 = severe negative energy balance animals. Libraries on the first flowcell were run at different concentrations (4.5–6.5 pM) to determine which concentration yielded optimum cluster yields.

### Transcriptional profile of cattle liver

**Figure 1:**
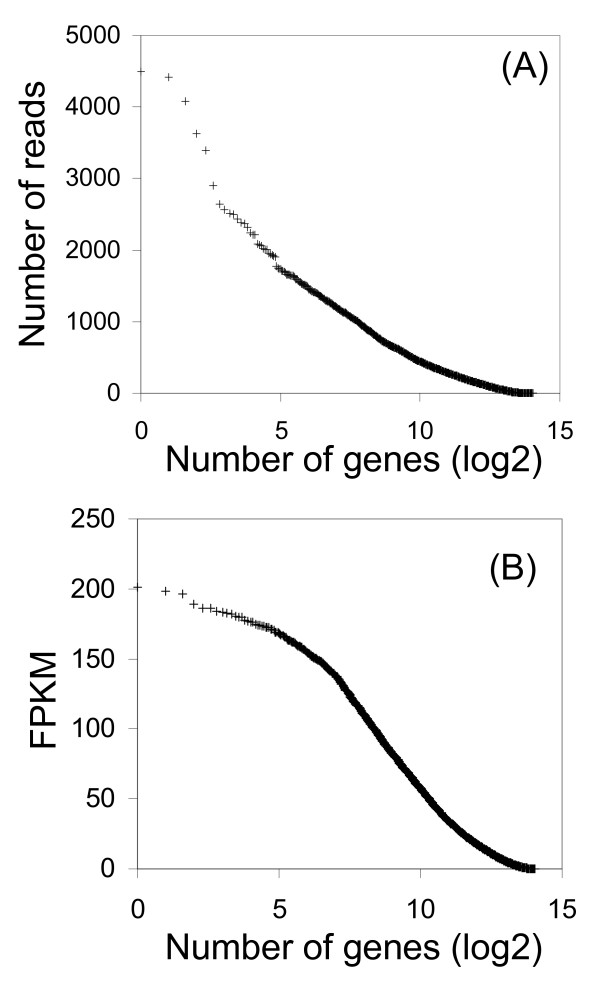
**Complexity of NEB cattle liver transcriptome.** (A) Average number of reads across 21 flowcell lanes aligning to genes. (B) Average FPKM values for genes across 21 flowcell lanes.

The lowest limit of detection was set to 5 or more uniquely aligned reads in at least one animal in either the SNEB or MNEB group. At this limit, 12,523 and 12,833 genes were detected as expressed in MNEB and SNEB animals respectively. This is similar to the number of human Ensembl genes detected in human (12,191) and mouse (11,201) liver tissue by polyA RNA-seq [25]. RNA-seq analysis of mouse and human liver by other groups showed that the 10 most highly expressed genes make up 20-40% of the mRNA pool whereas other tissue transcriptomes e.g. brain, kidney and testis are more complex with the 10 most highly expressed genes contributing to just 5-10% of the total mRNA [25]. We found that in the liver of cows in NEB, on average 16% of the total RNA-seq reads aligned to only 10 genes and 30% of the RNA-seq reads mapped to just 619 genes (Figure 1A) per lane. However, when we normalised for gene length by calculating values for fragments per kilobase of exon per million fragments mapped (FPKM) to get a better indication of relative transcript number, the top 10 most highly expressed genes represented only 0.77% of the transcript pool. There were highly abundant transcripts (FPKM >50) for approximately 1,200 genes in hepatic tissue analysed in the current study (Figure 1B). Depletion of very highly expressed long transcripts may be necessary to increase the sequencing coverage of less abundant shorter transcripts in mammalian liver RNA-seq libraries.

### Lane effect

**Figure 2:**
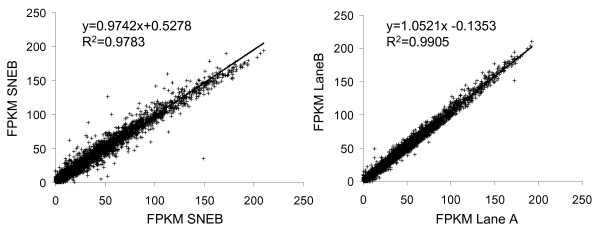
**Lane effect.** (**A**) Average FPKM values for each gene in SNEB animals correlated with average FPKM values for each gene in MNEB animals. (**B**) Average FPKM values for each gene for libraries 1, 2, 5, 6, 7, 10, 11, 12 run on lanes 1A, 3B, 3A, 2B, 4B, 7A, 7B, 1B respectively correlated with average FPKM values for each gene for libraries 1, 2, 5, 6, 7, 10, 11, 12 run on lanes 2A, 1C, 4A, 3C, 4C, 8A, 5A, 7B, 8C respectively.

To further determine whether the differential expression observed between MNEB and SNEB was due to a real difference between the groups and not simply random variation caused by running samples on different lanes (i.e. lane effect), correlation analysis was performed between FPKM values from the two treatment groups and also the same 9 libraries run as technical replicates on two different lanes (Figure 2). This showed that the difference in FPKM values (r^2^ = 0.9783 NEB vs. SNEB r^2^ = 0.9905 Lane A vs. Lane B) was predominately due to effect of treatment and not lane effect.

### Identification of SDE genes

DEseq is an R-based software package that was developed specifically for the identification of SDE genes from raw counts of sequence reads generated by RNA-seq analysis that uniquely align to genes. RNA-seq library 1 (corresponding to MNEB animal 1) was not included in these analyses as this animal subsequently became ill and a possible underlying infection may have skewed the gene expression data generated. DEseq analysis showed that 413 genes were SDE (FDR of 0.1%), 201 of which had a fold change of ≥2 (either up or down) between MNEB and SNEB animals (Additional file 2).

### Comparison of microarray and RNA-seq

We previously analysed the same MNEB and SNEB liver RNA samples using the Affymetrix 23 K bovine gene microarray platform and, of the 5,229 genes which were detected as expressed, 416 were identified as SDE by the puma method using the recommended cut-off P-like value of *P* <0.05 [11]. Of these, only 56 had fold changes of ≥2 (up or down) in SNEB animals. 55 of the 416 microarray and 413 RNA-seq SDE genes, and 27 of the 201 RNA-seq and 56 microarray ≥2-fold SDE genes, were detected as SDE by both platforms (Additional file 3). All genes detected as SDE on both platforms had fold changes in the same direction. This approximate comparison of the two data sets shows that, particularly for the lower fold changes, many genes were detected as SDE on one platform but not the other. However, RNA-seq detected expression of more than twice as many genes as the 23 K bovine microarray, and nearly four times as many ≥2-fold SDE genes.

### Physiology of SNEB model

Soon after calving in high-yielding dairy cows, nutritional and energetic demands can increase 4-fold within a single day as the animal undergoes a physiological upheaval to meet the demand by the mammary gland for substrates, particularly glucose, for milk production. The main changes of this upheaval are a reduction of insulin concentration in the blood, a reduction of lipogenesis in adipocytes, an increase in export of fats from adipose to liver, and a dramatic increase in gluconeogenesis in liver. Unlike non-ruminants, where glucose is absorbed via the small intestine, approximately 90% of all glucose in ruminants is synthesised by gluconeogenesis in the liver from volatile fatty acids (VFAs) derived from bacterial fermentation in the rumen. VFAs are absorbed across the rumen wall and into the hepatic portal vein which delivers them to the liver. The increase in hepatic gluconeogeneis at the onset of lactation leads to production of very high quantities of glucose. The glucose entry into the blood stream in top performing cows producing 90 kg milk per day has been estimated at 7.4 kg per day of which 4.4 kg will end up as lactose in milk [26].

In our model we were comparing two groups of lactating animals in the early post-calving period (13 or 14 days post-calving). One of these groups was in MNEB and the other was in a state of SNEB which was artificially induced by restricting feed and trebling the milking frequency. From this we hoped to identify novel genes and pathways which are important in SNEB in high-yielding dairy cows. Hormones, metabolites and aspects of liver composition measured at the time of tissue collection showed that, as predicted, relative to MNEB cows, SNEB cows had undergone significant (*P* <0.05) changes in energy balance (−3.6-fold), blood glucose (−1.5-fold), blood NEFA (+ 4.7-fold), blood BHB (+7.4), blood IGF1(−4.8-fold), liver TAG (+3.9-fold), liver glycogen (−10.4-fold). Blood insulin, oestradiol, urea and liver weight were not different between SNEB and MNEB animals [9]. The level of cholesterol was also lower in the SNEB cows (Additional file 4). These changes are widely known to occur in SNEB cows [10] where there is an increase in the export of NEFAs from adipose to the liver which exceeds the normal oxidative capacity of the liver. This results in an increase in ketone bodies (BHB) in the blood resulting from partially oxidised NEFAs, and an accumulation of TAGs in the liver. In addition, IGF1 levels drop as hepatocytes become refractory to growth hormone (GH). Under normal conditions, growth hormone (GH) is released from the anterior pituitary in the brain, binds to the growth hormone receptor (GHR) on the surface of hepatocytes, and this binding triggers a signalling cascade that leads to increased IGF1 synthesis by the liver for export to the blood. In NEB cows the liver becomes refractory to GH and this results in low circulating IGF1 [10].

### Over-represented KEGG pathways

**Figure 3:**
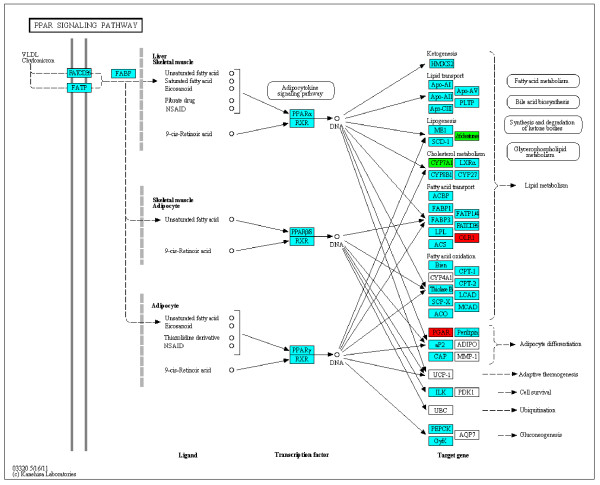
**Changes in hepatic gene expression in the KEGG PPAR signalling pathway in SNEB compared to MNEB cows.** Blue boxes are genes detected by RNA-seq as expressed but not SDE, red boxes are SDE genes with reduced expression in SNEB animals, green boxes are SDE genes with increased expression in SNEB animals.

**Figure 4:**
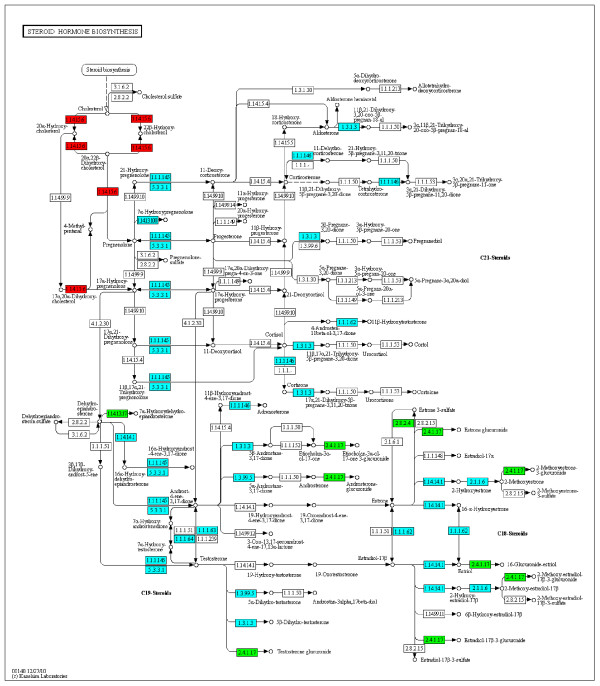
**Changes in hepatic gene expression in the KEGG Steroid hormone biosynthesis pathway in SNEB compared to MNEB cows.** Blue boxes are KEGG enzymes coded for by genes detected by RNA-seq as expressed but not SDE, red boxes are KEGG enzymes coded for by SDE genes with reduced expression in SNEB animals, green boxes are KEGG enzymes coded for by SDE genes with increased expression in SNEB animals. KEGG enzyme 1.14.15.6 is coded for by HSA CYP11A1, KEGG enzyme 1.14.13.17 is coded for by HSA *CYP7A1*, KEGG enzyme 2.4.1.17 is coded for by HSA *UG2A1*, KEGG enzyme 2.8.2.4 is coded for by HSA *SULT1E1*.

In our RNA-seq study, nine KEGG pathways were associated with ≥2-fold SDE genes most of these were related to metabolism of fats (Table 2, Figures 3, 4, Additional file 5). Pathways related to the metabolism of carbohydrates were only over-represented when SDE genes with <2-fold change were included in the pathway analysis, (Table 3, Additional file 6). This suggests that in our model, at the level of transcription in the liver, to compensate for the reduced supply of VFAs from the liver due to feed restriction, the major changes in hepatic gene expression in SNEB cows were related to the alternative supply of carbon from fat for gluconeogenesis. The most interesting finding from our RNA-seq analysis was that ‘Steroid hormone biosynthesis’, a process which is normally exclusive to the gonads and adrenal glands, was the KEGG pathway most significantly associated with ≥2-fold SDE genes in SNEB cow liver. Details of this and other selected pathways are discussed below in the context of the expected physiological changes in the liver of SNEB cows.

**Table 2:** Summary of KEGG pathways associated with ≥2 fold SDE genes

**Pathway code**	**Pathway name**	**Over represented***P***value**
00140	Steroid hormone biosynthesis	0.000163
03320	PPAR signalling pathway	0.000903
04976	Bile secretion	0.000960
04977	Vitamin digestion and absorption	0.003082
04975	Fat digestion and absorption	0.004934
02010	ABC transporters	0.006003
00592	α-linolenic acid metabolism	0.006639
01040	Biosynthesis of unsaturated fatty acids	0.022054
00830	Retinol metabolism	0.031908

Pathway maps for KEGG pathways associated with ≥2-fold SDE genes (FDR0.1%) that are not included in the main text are in Additional file 5.

**Table 3:** Summary of KEGG pathways associated with all SDE genes

**Pathway code**	**Pathway name**	**Over represented***P***value**
04976	Bile secretion	0.001483
00140	Steroid hormone biosynthesis	0.002046
04950	Maturity onset diabetes of the young	0.004947
00910	Nitrogen metabolism	0.007765
00061	Fatty acid biosynthesis	0.011248
05219	PPAR signalling pathway	0.018112
03320	Bladder cancer	0.018941
04910	p53 signalling pathway	0.020756
04115	Insulin signalling pathway	0.021653
04977	Vitamin digestion and absorption	0.026059
00592	α-linolenic acid metabolism	0.030405
04975	Fat digestion and absorption	0.034363
04080	Neuroactive ligand-receptor interaction	0.040178

Pathway maps for KEGG pathways associated with all SDE genes (FDR 0.1.%) are in Additional file 6.

### Carbohydrate metabolism

The 1.5-fold drop in blood glucose in SNEB cows would suggest decreased gluconeogenesis in the liver of these animals. However, only a minor decrease (≤2-fold) of pyruvate carboxylase (PYK (KEGG pathway map) = *PKLR* (SDE gene list), one of the major rate limiting enzymes in gluconeogenesis [27] was identified in the KEGG ‘Insulin signalling pathway’ (Additional files 2 and 6). The KEGG pathway ‘Glycolysis/gluconeogeneis’ was not identified as over-represented. This indicates that, in feed-restricted SNEB animals, there was only a slight decrease in the rate of gluoneogenesis and that demands for carbon for this process were mostly being met by oxidation of NEFAs transported from adipose. There were no gene pathways to related to the 10-fold drop in liver glycogen in observed in SNEB cows even though genes coding for enzymes involved in glycogen metabolism (e.g. PYG, PHK) were detected as expressed in the KEGG ‘Insulin signalling pathway’ (Additional file 6). Major changes in transcription of genes controlling glycogenolysis may have occurred prior to collection of liver tissue for RNA extraction.

### Uncoupling of the GH/IGF1 axis

Although pathways related to the GH/IGF1 axis were not identified as overrepresented among the SDE genes identified by RNA-seq, several genes within in this pathway were detected as SDE (*GHR −*1.6-fold, *IGFALS −*5-fold, *IGF1* -5-fold) (Additional file 2). The KEGG ‘p53 signalling pathway’, which is related to stress and includes *IGF1*, was overrepresented (Table 3). Hepatic expression of *IGF1, GHR* and *IGFALS* is well known to be reduced in SNEB cows and has been previously described [9,11]. Interestingly, for the same total RNA samples, *IGF1* was detected as SDE by both RNA-seq and qPCR but not by microarray (Additional file 3) [11], even though the amount of blood *IGF1*, which is made mainly in the liver, was significantly reduced in SNEB cows. Microarray also failed to detect *GHR* as SDE. However, our microarray study did, whereas our RNA-seq study did not, identify *IGFBP2* and *IGFBP3* (genes encoding *IGF1* binding proteins) as SDE (≤2-fold) in SNEB cow liver.

### TAG accumulation and increased blood NEFA

Increased TAG accumulation in the liver was reflected by overrepresentation of the KEGG ‘PPAR signalling pathway’ (Table 2, Figure 3) as angiopoietin-like 4 gene (PGAR (KEGG pathway map, Figure 3) = *ANGPTL4* (SDE gene list, Additional file 2) was upregulated in this pathway. Angiopoietin-like protein 4 has emerged as a key regulator of plasma cholesterol, triglyceride, and NEFA concentrations and, in cattle, is most highly expressed in the liver and adipose [28]. The upregulation of *ANGPTL4* due to fasting, corresponding to the SNEB condition, was also reported in adipose tissues of lactating goats [29]. Upregulation of *ANGPTL4*, which is stimulated by PPAR agonists, leads to inhibition of lipoprotein lipase activity in adipose, reduced VLDL-TAG utilisation, and increased lipolysis. This gene was previously identified by microarray to be up-regulated in the liver of induced ketosis cows [10] and SNEB cows [11] and it was proposed that increased *ANGPTL4* serves as a signal for lipolysis and contributes to sustained release of NEFA and lipid accumulation in the liver [10].

### Oxidation of NEFAs and ketogenesis

Due to the higher amount of NEFAs in the blood of SNEB animals, we were expecting changes in expression of genes to reflect increased oxidation of fatty acids in SNEB animals. However, although eight of the nine fatty acid oxidation genes in the KEGG PPAR signalling pathway were detected, none were SDE (Additional file 2). Acetyl-CoA carboxylase beta (*ACACB*) which is thought to control fatty acid oxidation was SDE (upregulated 3-fold) in RNA-seq but not picked up in the pathway analysis. KEGG PPAR signalling pathway is also involved in ketogenesis but, although the main gene involved in this (*HMGCS2*) was detected, it was not identified as SDE. This may be because at the time of slaughter there was little difference in the rate of oxidation and ketogenesis between the two groups, or that there were differences but not at the level of transcription. *OLR1* (oxidized low density lipoprotein (lectin-like) receptor 1) is included in the KEGG PPAR signalling pathway and was detected as upregulated (although read counts were very low for this gene). The protein encoded by *OLR1* binds, internalizes and degrades oxidized low-density lipoprotein (oxLDL) and is thought to have a role in lowering systemic oxidative stress by removal of oxLDL from blood.

### Impaired PUFA synthesis

The most SDE gene we identified by RNA-seq was *FADS2* (previously named Δ6 desaturase) which was down-regulated in SNEB animals (Figure 3, Additional files 2) and featured in 3 of the nine ≥2-fold KEGG pathways (α-Linolenic acid metabolism, Biosynthesis of unsaturated fatty acids, and PPAR signalling) (Additional file 5). *FADS1* was also shown to be down-regulated in SNEB animals (Additional files 2). *FADS1* and *FADS2* were previously identified by microarray as downregulated in liver of cows with induced clinical ketosis (days 9–14 post partum) [10]. However, in our microarray study, *FADS2*, but not *FADS1*, was identified as SDE. *FADS2* catalyses the initial desaturation step of α-linolenic acid (C18:3) and linoleic acid (C18:2) in the enzyme cascade that ultimately leads, via *FADS1*, to synthesis of the long chain polyunsaturated fatty acids (LC-PUFAs) arachidonic acid (C20:4) and eicosapentaenoic acid (C20:5). Synthesis of LC-PUFAs arachidonic acid and eicosapentaenoic acid mainly occurs in the liver and adipose [30] and suppression of their synthesis in SNEB cows may contribute to the poor fertility in high-yielding dairy herds. It was recently reported that polymorphisms in *FADS1* and *FADS2* were associated with reduced serum arachidonic levels in humans [31]. Also, deletion of *FADS2* gene expression in mice leads to sterility in both male and female mice without affecting their viability [32].

### Unexpected overrepresentation of KEGG pathway ‘steroid hormone biosynthesis’

One of the most interesting findings of our RNA-seq study was that the KEGG pathway ‘Steroid hormone biosynthesis’ (Table 2, Figure 4) showed the most significant association with ≥2-fold SDE genes in SNEB animals. This is unexpected as, under normal conditions, steroid hormone biosynthesis occurs almost exclusively in the adrenal glands and gonads, whereas liver is the site for steroid hormone inactivation. Four genes were changed in this pathway. *CYP11A1* was up-regulated while *UGT2a1*, *SULTE1* and *CYP7A1* were down-regulated. *CYP11A1* was also identified as SDE in SNEB cow liver in our microarray study [11] but not in the liver of induced ketosis cows [10]. *CYP7A1*, *UGT2A1* and *SULT1E1* were not identified as SDE in either of those studies. *CYP7A1* catalyses the rate limiting step of conversion of cholesterol to bile acids and was also included in the KEGG pathway ‘Bile secretion’ which was overrepresented in the ≥2-fold SDE genes identified by RNA-seq (Table 2, Additional file 5). *UGT2A1* and *SULT1E1* inactivate oestrogens by glucuronidation and sulphation respectively, prior to their excretion. The down-regulation of these genes in the liver of SNEB animals indicates that bile synthesis and inactivation of oestrogens in liver is reduced in SNEB possibly to conserve cholesterol which was reduced in SNEB animals (Additional file 4). This also suggests that lower blood concentration of oestrodiol in preovulatory high yielding dairy cows may not be due to increased oestrogen inactivation in liver.

### CYP11A1, a novel gene in metabolic adaptations to post-partum bovine SNEB

Although we detected upregulation of *CYP11A1* in liver of SNEB cows in our microarray study, ‘Steroid hormone biosynthesis’ was not associated with the microarray SDE genes, probably because *CYP7A1*, *UGT2A1* and *SULTE1* were not identified as SDE, so the implications of upregulation of *CYP11A1* in SNEB cows were not discussed [11]. To the knowledge of the authors, other than in our own microarray study, expression of *CYP11A1* has not been previously described in adult cow liver. Other workers found *CYP11A1* to be undetectable by qPCR in the liver of adult cows and consequently used liver as a negative control for expression studies of *CYP11A1* in granulosa [33]. However, *CYP11A1* does appear to be expressed in foetal and juvenile cows [34]. Up-regulation of this gene is unexpected as it catalyses the initial step of conversion of cholesterol into pregnenolone which is then converted by *CYP17A1* to dehydroepiandrosterone (DHEA). DHEA is the precursor for a number of steroid hormones including oestrogen. Although other tissues, such as brain and foetal rat liver, also express the enzymes for steroid biosynthesis [35] these enzymes are not normally expressed in adult mammalian liver. Unexpected up-regulation of CYP11A1 was also observed in vitro in human hepatocytes after treatment with adenovirus expressing PGC-1 alpha [35] which is a key integrator of many of the signalling pathways that are induced in the liver and muscle upon fasting. In that study, *CYP17A1* was also upregulated and the authors proposed that DHEA (or one of its metabolites) in liver could be involved in a novel hepatic signalling pathway or may serve to protect hepatocytes from the increased oxidative state brought on by fasting. DHEA has been shown to protect endothelial cells from apoptosis. They also suggested that *CYP11A1* expression in feed restricted hepatocytes may be involved in regulation of cholesterol homeostasis as *CYP11A1* catalyses modifications on cholesterol to produce oxysterols which are known ligands for liver X receptors (LXRs). LXRs regulate cholesterol homeostasis and lipid metabolism. It would therefore be interesting to see if DHEA levels in SNEB cow liver are elevated. In both our microarray and RNA-seq study, SDE genes included in the IPA canonical signalling pathway ‘*LXR/RXR* activation’ were changed (*ABCG8*, *ACACA* (downregulated); *ARG*, *CCL2*, *IL1RN*, *RXRG* (upregulated)). In our RNA-seq data, many of the genes, including *CYP17A1*, in the KEGG pathway ‘Steroid hormone biosynthesis’ were not detected as expressed (Additional file 5). This may either be due to the fact that these genes are duplicated in the genome and thus reads were eliminated because they mapped to more than one locus (e.g. *CYP17A1* (human gene name) has an exact copy (*LOC784256*) at another position in the *Bos taurus* genome), transcript abundance was too low for detection, or that they are not expressed in the liver thus preventing complete synthesis of steroid hormones in this organ.

## Conclusions

RNA-seq analysis showed that the major changes at the level of transcription in the liver of SNEB cows were related to fat metabolism. Unexpectedly, one of the most significantly changed biological pathways was ‘Steroid hormone biosynthesis’ where *CYP11A1* was upregulated. In adult cows, this gene is normally only expressed in the adrenal glands and ovaries and not liver. *CYP11A1* is a potentially novel gene that may play a key role in metabolic adaptations to NEB in high-yielding dairy cows.

## Materials and methods

### Animal model

The animal model employed in this study has been described previously [9], and all procedures were carried out under license in accordance with the European Community Directive 86-609-EC. The nutritional and lactational management regime employed were designed to create significant divergence in the energy balance (EB) profiles of the cows in early lactation. In brief, multiparous Holstein-Friesian cows (n = 24) were blocked 2 wk prior to expected calving date according to parity, body condition score (BCS), and previous lactation yield (average lactation 6,477 ± 354 kg) and randomly allocated to mild (MNEB, n = 12) or severe (SNEB, n = 12) NEB groups. MNEB cows were fed *ad libitum* grass silage and 8 kg/day concentrates and milked once daily; SNEB cows were fed 25 kg/day silage and 4 kg/day concentrate and milked three times daily. Measurements of BCS and EB were used to select cows that showed extremes in EB from each group (MNEB, n = 5; SNEB, n = 6). Cows were slaughtered on days 6–7 of the first follicular wave after calving (mean number of days postpartum: MNEB mean 13.6 ± 0.75, range 11–15; SNEB mean 14.3 ± 0.56, range 13–16), based on daily transrectal ultrasonography.

### Collection of liver tissue for RNA and TAG analysis

The entire liver was removed within 15–30 min after slaughter and weighed. Samples weighing approximately 1 g were dissected, rinsed in RNase-free phosphate buffer, snap-frozen in liquid nitrogen, and stored at −80°C. For triacylglyceride (TAG) analysis, total lipids were extracted from 50 mg samples of liver as previously described [9].

### Blood sampling and metabolite assays

Stabilized (EDTA-treated) whole blood samples were collected on the day of slaughter by jugular venipuncture for haematological analysis. Blood samples were analyzed for glucose, NEFA, β-hydroxybutyrates (BHB), and urea using appropriate kits and an ABX Mira autoanalyser (ABX Mira, Cedex, France). All metabolite assay coefficients of variation were low and typically <5%.

### RNA extraction and quality analysis

Total RNA was prepared from 100–200 mg of fragmented frozen liver tissue using the TRIzol reagent (Sigma-Aldrich, Dorset, UK). Tissue samples were homogenized in 3 ml of TRIzol reagent and chloroform and subsequently precipitated using isopropanol (Sigma Chemical, Wicklow, Ireland). RNA samples were stored at −80°C. 20 μg of total RNA from each sample for genomic DNA contamination was treated with the RNase-free DNase set (QIAGEN, Crawley, West Sussex, UK) and purified it using the RNeasy mini kit in accordance with guidelines supplied (QIAGEN, Crawley, West Sussex, UK). RNA quality and quantity were assessed using automated capillary gel electrophoresis on a Bioanalyzer 2100 with RNA 6000 Nano Labchips according to manufacturer’s instructions (Agilent Technologies Ireland, Dublin, Ireland). Samples of RNA had 28 S/18 S ratios ranging from 1.8 to 2.0 and RNA integrity number values of between 8.0 and 10.0.

### Preparation and sequencing of polyA mRNA-seq ilumina libraries

polyA RNA was isolated from 5–10 ug total RNA with oligo(dT) beads using two rounds of oligo-dT purification. 5–10 ug RNA was fragmented with zinc fragmentase (Applied Biosystems, Warrington, UK), first strand cDNA synthesis was performed using the Invitrogen random hexamer primers and SuperScript II (Invitrogen)**,** second strand synthesis was performed using Invitrogen DNA Polymerase 1 (Invitrogen). End repair and polyadenylation were performed using NEB Next Tailing Module (New England Biolabs). ® End Repair Module and NEB Next dA- Illumina single read adapters were ligated to blunt ended, polyadenylated fragments with a NEB Quick ligation kit (New England Biolabs). Adapter-ligated cDNA fragment libraries were run on an Illumina GAII using version 3 sequencing kits and version 3 single read cluster generation kits.

### Read alignment

The reads from each of the lanes were aligned separately to the BCM4 genome assembly [23], using the ultrafast short read aligner, Bowtie (version 0.12.3) [24]. Fastq output files from the sequencer were used as input, specifying the following options: quality scores are ASCII characters equal to the Phred quality scores plus 64 (−−Solexa1.3-quals); the maximum number of mismatches allowed in the first 28 bases is 2 (−n 2, –l 28); suppressing all alignments for any read that has more then 1 reportable alignment (−m 1); retained alignments were reported in SAM format (−S).

### Read abundance calculation

The abundance of mRNAs for all annotated genes from the ENSEMBL v59 annotation of the bovine genome [36], was calculated using the software package HTseq (version 0.4.4p6) (http://sourceforge.net/projects/htseq/). The script HTseq-count provided with this package was used to count the number of reads that mapped to each annotated gene, allowing, in some cases, for reads to partially overlap with the exons and still be counted for that gene (i.e. -m union). As our sequencing analysis included technical replicates of individual samples, we summed the counts for all these lanes, resulting in a single count for each gene for each sample. The counts for all the samples were collated into one file and any gene with fewer than 5 reads in all samples was excluded from the subsequent statistical analysis of differential gene expression.

### Identification of SDE genes and pathway analysis

We identified SDE using DEseq (version 1.1.11) [21]. DEseq uses a generalisation of the Poisson model, the negative binomial distribution, to model biological and technical variance and test for differential expression between two experimental conditions. As there are nearly 30,000 genes annotated in the bovine genome, the statistical tests in each analysis were corrected for multiple testing using the Benjamini and Hochberg (BH) method [37] as implemented in R (version 2.12.0). All genes that were found to be SDE between the two experimental conditions (at an FDR p-value cut-off of 0.1) were retained for further analysis.

GOseq and KEGG pathway analysis tools were used to identify biological pathways that were significantly enriched in the data set of SDE genes (Table 2, Additional file 3). To facilitate this, reads were firstly converted to their human orthologs. GOseq specifically adjusts for the higher abundance of reads from long or highly expressed genes in RNA-seq experiments when assessing a gene list for over-representation of biological functions [22]. Genes were mapped to the KEGG database [38] for pathway analysis using GOseq [22].

## Competing interests

The authors declare that they have no competing interests.

## Authors’ contributions

MSM generated the RNA-seq libraries, co-ordinated the sequencing runs, helped with retrieval and analysis of data, carried out detailed interpretation of the pathway analysis, and prepared the main manuscript. SW extracted the RNA from NEB liver samples, conceived the idea of performing RNA-seq on the NEB RNA samples and contributed to the biological interpretation of the data and manuscript preparation. DL contributed to the bioinformatics of differential gene expression analysis and pathway analysis and writing and editing of the manuscript. DK, and DM co-ordinated tissue collection and RNA extraction from the NEB model, conducted metabolite studies on the model and contributed to the biological interpretation of the data and writing and editing of the manuscript. CC set up and performed the majority of the bioinformatics on the sequencing runs including data retrieval, read alignments, differential gene expression analysis, KEGG pathway analysis, data interpretation and co-wrote the main manuscript with MSM. All authors read and approved the final manuscript.

## Supplementary Material

Additional file 1 Table of number and assignment of reads for each flowcell lane. Click here for file

Additional file 2 Table SDE genes and their fold-change. Click here for file

Additional file 3 Comparison of microarray and RNA-seq SDE genes. Click here for file

Additional file 4 Graph of effect of SNEB compared to MNEB on blood concentrations of major NEB associated metabolites 1–14 days post-calving. Click here for file

Additional file 5 Maps of KEGG pathways (associated with ≥2 fold (FDR 0.1%) SDE genes) overrepresented in SNEB animals. Click here for file

Additional file 6 Maps of KEGG pathways associated with all SDE (FDR 0.1%) genes. Click here for file

## References

[B1] Wathes DC Cheng ZR Chowdhury W Fenwick MA Fitzpatrick R Morris DG Patton J Murphy JJ Negative energy balance alters global gene expression and immune responses in the uterus of postpartum dairy cows Physiol Genomics 2009 39 1 1 13 10.1152/physiolgenomics.00064.2009 19567787 PMC2747344

[B2] Drackley JK ADSA Foundation Scholar Award. Biology of dairy cows during the transition period: the final frontier? J Dairy Sci 1999 82 11 2259 2273 10.3168/jds.S0022-0302(99)75474-3 10575597

[B3] Pullen DL Liesman JS Emery RS A species comparison of liver slice synthesis and secretion of triacylglycerol from nonesterified fatty-acids in media J Anim Sci 1990 68 5 1395 1399 2365651 10.2527/1990.6851395x

[B4] Reynolds CK Aikman PC Lupoli B Humphries DJ Beever DE Splanchnic metabolism of dairy cows during the transition from late gestation through early lactation Journal of Dairy Science 2003 86 4 1201 1217 10.3168/jds.S0022-0302(03)73704-7 12741545

[B5] Morris DG Waters SM McCarthy SD Patton J Earley B Fitzpatrick R Murphy JJ Diskin MG Kenny DA Brass A Pleiotropic effects of negative energy balance in the postpartum dairy cow on splenic gene expression: repercussions for innate and adaptive immunity Physiol Genomics 2009 39 1 28 37 10.1152/physiolgenomics.90394.2008 19567785 PMC2747343

[B6] Pickett MM Piepenbrink MS Overton TR Effects of propylene glycol or fat drench on plasma metabolites, liver composition, and production of dairy cows during the periparturient period J Dairy Sci 2003 86 6 2113 2121 10.3168/jds.S0022-0302(03)73801-6 12836948

[B7] Wathes DC Fenwick M Cheng Z Bourne N Llewellyn S Morris DG Kenny D Murphy J Fitzpatrick R Influence of negative energy balance on cyclicity and fertility in the high producing dairy cow Theriogenology 2007 68 s232 s241 17475319 10.1016/j.theriogenology.2007.04.006

[B8] Loor JJ Genomics of metabolic adaptations in the peripartal cow Animal 2010 4 7 1110 1139 10.1017/S1751731110000960 22444613

[B9] Fenwick MA Fitzpatrick R Kenny DA Diskin MG Patton J Murphy JJ Wathes DC Interrelationships between negative energy balance (NEB) and IGF regulation in liver of lactating dairy cows Domest Anim Endocrinol 2008 34 1 31 44 10.1016/j.domaniend.2006.10.002 17137745

[B10] Loor JJ Everts RE Bionaz M Dann HM Morin DE Oliveira R Rodriguez-Zas SL Drackley JK Lewin HA Nutrition-induced ketosis alters metabolic and signalling gene networks in liver of periparturient dairy cows Physiol. Genomics 2007 32 105 116 10.1152/physiolgenomics.00188.2007 17925483

[B11] McCarthy SD Waters SM Kenny DA Diskin MG Fitzpatrick R Patton J Wathes DC Morris DG Negative energy balance and hepatic gene expression patterns in high-yielding dairy cows during the early postpartum period: a global approach Physiol Genomics 2010 42A 3 188 199 10.1152/physiolgenomics.00118.2010 20716645 PMC3008362

[B12] Huang W Khatib H Comparison of transcriptomic landscapes of bovine embryos using RNA-Seq BMC Genomics 2010 11 711 10.1186/1471-2164-11-711 21167046 PMC3019235

[B13] Mortazavi A Williams BA McCue K Schaeffer L Wold B Mapping and quantifying mammalian transcriptomes by RNA-Seq Nat Methods 2008 5 7 621 628 10.1038/nmeth.1226 18516045 PMC13303166

[B14] Wang Z Gerstein M Snyder M RNA-Seq: a revolutionary tool for transcriptomics Nat Rev Genet 2009 10 1 57 63 10.1038/nrg2484 19015660 PMC2949280

[B15] Kim TK Hemberg M Gray JM Costa AM Bear DM Wu J Harmin DA Laptewicz M Barbara-Haley K Kuersten S Widespread transcription at neuronal activity-regulated enhancers Nature 2010 465 7295 182 187 10.1038/nature09033 20393465 PMC3020079

[B16] Sultan M Schulz MH Richard H Magen A Klingenhoff A Scherf M Seifert M Borodina T Soldatov A Parkhomchuk D A global view of gene activity and alternative splicing by deep sequencing of the human transcriptome Science 2008 321 5891 956 960 10.1126/science.1160342 18599741

[B17] Cánovas A Rincon G Islas-Trejo A Wickramasinghe S Medrano JF (2010) SNP discovery in the bovine milk transcriptome using RNA-Seq technology Mamm Genome 2010 21 11–12 592 598 21057797 10.1007/s00335-010-9297-z PMC3002166

[B18] Maher CA Palanisamy N Brenner JC Cao X Kalyana-Sundaram S Luo S Khrebtukova I Barrette TR Grasso C Yu J Chimeric transcript discovery by paired-end transcriptome sequencing Proc Natl Acad Sci U S A 2009 106 30 12353 12358 10.1073/pnas.0904720106 19592507 PMC2708976

[B19] Oshlack A Robinson MD Young MD From RNA-seq reads to differential expression results Genome Biol 2010 11 12 220 10.1186/gb-2010-11-12-220 21176179 PMC3046478

[B20] Garber M Grabherr MG Guttman M Trapnell C Computational methods for transcriptome annotation and quantification using RNA-seq Nat Methods 2011 8 6 469 477 10.1038/nmeth.1613 21623353

[B21] Anders S Huber W Differential expression analysis for sequence count data Genome Biol 2010 11 10 R106 10.1186/gb-2010-11-10-r106 20979621 PMC3218662

[B22] Young MD Wakefield MJ Smyth GK Oshlack A Gene ontology analysis for RNA-seq: accounting for selection bias Genome Biol 2010 11 2 R14 10.1186/gb-2010-11-2-r14 20132535 PMC2872874

[B23] Elsik CG Tellam RL Worley KC Gibbs RA Muzny DM Weinstock GM Adelson DL Eichler EE Elnitski L Guigo R The genome sequence of taurine cattle: a window to ruminant biology and evolution Science 2009 324 5926 522 528 19390049 10.1126/science.1169588 PMC2943200

[B24] Langmead B Trapnell C Pop M Salzberg SL Ultrafast and memory-efficient alignment of short DNA sequences to the human genome Genome Biol 2009 10 3 R25 10.1186/gb-2009-10-3-r25 19261174 PMC2690996

[B25] Ramskold D Wang ET Burge CB Sandberg R An abundance of ubiquitously expressed genes revealed by tissue transcriptome sequence data PLoS Comput Biol 2009 5 12 e1000598 10.1371/journal.pcbi.1000598 20011106 PMC2781110

[B26] Aschenbach JR Kristensen NB Donkin SS Hammon HM Penner GB Gluconeogenesis in dairy cows: the secret of making sweet milk from sour dough Iubmb Life 2010 62 12 869 877 10.1002/iub.400 21171012

[B27] Pershing RA Moore SD Dinges AC Thatcher WW Badinga L Short communication: hepatic gene expression for gluconeogenic enzymes in lactating dairy cows treated with bovine somatotropin Journal of Dairy Science 2002 85 3 504 506 10.3168/jds.S0022-0302(02)74101-5 11949852

[B28] Mamedova LK Robbins K Johnson BJ Bradford BJ Tissue expression of angiopoietin-like protein 4 in cattle J Anim Sci 2010 88 1 124 130 10.2527/jas.2009-2258 19783696

[B29] Faulconnier Y Chilliard Y Montazer Torbati MB Leroux C The transcriptomic profiles of adipose tissues are modified by feed deprivation in lactating goats Comparative Biochemistry and Physiology, Part D 2011 6 2 139 149 10.1016/j.cbd.2010.12.002 21256818

[B30] Jacobi SK Lin X Corl BA Hess HA Harrell RJ Odle J Dietary arachidonate differentially alters desaturase-elongase pathway flux and gene expression in liver and intestine of suckling pigs J Nutr 2011 141 4 548 553 10.3945/jn.110.127118 21310868

[B31] Lattka E Illig T Koletzko B Heinrich J Genetic variants of the FADS1 FADS2 gene cluster as related to essential fatty acid metabolism Curr Opin Lipidol 2010 21 1 64 69 10.1097/MOL.0b013e3283327ca8 19809313

[B32] Stoffel W Holz B Jenke B Binczek E Gunter RH Kiss C Karakesisoglou I Thevis M Weber AA Arnhold S Delta6-desaturase (FADS2) deficiency unveils the role of omega3- and omega6-polyunsaturated fatty acids EMBO J 2008 27 17 2281 2292 10.1038/emboj.2008.156 19172737 PMC2529369

[B33] Vanselow J Spitschak M Nimz M Furbass R DNA methylation is not involved in preovulatory down-regulation of CYP11A1, HSD3B1, and CYP19A1 in bovine follicles but may have a role in permanent silencing of CYP19A1 in large granulosa lutein cells Biol Reprod 2010 82 2 289 298 10.1095/biolreprod.109.079251 19794152

[B34] Bovine gene atlashttp://bovine atlas.msstate.edu

[B35] Grasfeder LL Gaillard S Hammes SR Ilkayeva O Newgard CB Hochberg RB Dwyer MA Chang CY McDonnell DP Fasting-induced hepatic production of DHEA is regulated by PGC-1alpha, ERRalpha, and HNF4alpha Mol Endocrinol 2009 23 8 1171 1182 10.1210/me.2009-0024 19389810 PMC2718748

[B36] Flicek P Aken BL Ballester B Beal K Bragin E Brent S Chen Y Clapham P Coates G Fairley S Ensembl's 10th year Nucleic Acids Res 2010 38 D557 D562 10.1093/nar/gkp972 19906699 PMC2808936

[B37] Benjamini Y Hochberg Y Controlling the false discovery rate - a practical and powerful approach to multiple testing Journal of the Royal Statistical Society Series B-Methodological 1995 57 1 289 300

[B38] KEGG pathway data basehttp://www.genome.jp/kegg/pathway.html

