# Supplementary material for: RNA-seq analysis of differential gene expression in liver from lactating dairy cows divergent in negative energy balance
Source: BMC Genomics. 2012 May 20;13:193. doi: 10.1186/1471-2164-13-193 (PMC3465249; doi:10.1186/1471-2164-13-193)
Supplement: Additional file 1 — Table of number and assignment of reads for each flowcell lane. [file 1471-2164-13-193-S1.doc]

**Additional File 1** Assignment of reads for each lane.

| **FlowCell** | **Lane** | **Sample** | **Group** | **Raw Reads** | **Failed to align** | **Aligned to multiple positions** | **Accepted Alignments** | **Reads removed because of possible PCR bias** | **Reads retained** | **Reads Mapped to annotated exons** | **Reads mapped to exon-exon junctions** |
| --- | --- | --- | --- | --- | --- | --- | --- | --- | --- | --- | --- |
| 1 | 1 | 1 | MNEB | 15,562,688 | 3,239,348 | 2,579,369 | 9,743,971 | 5,566,128 | 4,177,843 | 2,211,875 | 317,205 |
| 1 | 2 | 1 | MNEB | 15,760,306 | 3,198,686 | 2,633,205 | 9,928,415 | 5,688,955 | 4,239,460 | 2,224,880 | 327,114 |
| 1 | 3 | 5 | MNEB | 15,603,465 | 3,519,875 | 2,488,576 | 9,595,014 | 5,336,584 | 4,258,430 | 2,147,404 | 272,662 |
| 1 | 4 | 5 | MNEB | 6,388,721 | 1,389,362 | 1,019,957 | 3,979,402 | 1,867,863 | 2,111,539 | 1,221,833 | 142,028 |
| 1 | 5 | 10 | SNEB | 15,611,292 | 3,181,301 | 2,590,556 | 9,839,435 | 5,382,352 | 4,457,083 | 2,304,002 | 289,430 |
| 1 | 7 | 10 | SNEB | 15,743,036 | 3,212,743 | 2,612,481 | 9,917,812 | 5,430,993 | 4,486,819 | 2,306,798 | 287,241 |
| 1 | 8 | 10 | SNEB | 14,433,338 | 2,941,620 | 2,369,327 | 9,122,391 | 4,918,264 | 4,204,127 | 2,241,342 | 269,937 |
| 2 | 1 | 12 | SNEB | 11,224,326 | 2,381,028 | 1,949,177 | 6,894,121 | 3,882,248 | 3,011,873 | 1,658,352 | 218,243 |
| 2 | 2 | 6 | MNEB | 12,276,914 | 2,784,074 | 1,935,876 | 7,556,964 | 4,162,552 | 3,394,412 | 1,807,515 | 279,983 |
| 2 | 3 | 2 | MNEB | 13,188,370 | 2,661,447 | 2,235,379 | 8,291,544 | 4,361,992 | 3,929,552 | 2,002,035 | 278,150 |
| 2 | 4 | 7 | SNEB | 13,547,188 | 2,822,735 | 2,667,321 | 8,057,132 | 4,171,044 | 3,886,088 | 2,009,696 | 263,442 |
| 2 | 5 | 9 | SNEB | 13,555,104 | 2,561,920 | 2,366,808 | 8,626,376 | 4,922,855 | 3,703,521 | 1,869,837 | 231,470 |
| 2 | 7 | 11 | SNEB | 13,181,523 | 2,667,150 | 2,259,413 | 8,254,960 | 4,518,092 | 3,736,868 | 1,943,947 | 287,517 |
| 2 | 8 | 4 | MNEB | 12,730,893 | 2,659,701 | 2,029,408 | 8,041,784 | 4,504,898 | 3,536,886 | 1,774,011 | 240,343 |
| 3 | 1 | 2 | MNEB | 12,042,460 | 2,488,591 | 1,908,460 | 7,645,409 | 4,030,278 | 3,615,131 | 2,003,287 | 231,920 |
| 3 | 2 | 3 | MNEB | 13,926,450 | 2,792,016 | 2,387,701 | 8,746,733 | 5,023,823 | 3,722,910 | 1,994,076 | 259,727 |
| 3 | 3 | 6 | MNEB | 14,213,294 | 2,985,164 | 2,249,916 | 8,978,214 | 5,092,229 | 3,885,985 | 2,144,462 | 296,221 |
| 3 | 4 | 7 | SNEB | 14,363,214 | 2,954,315 | 2,764,699 | 8,644,200 | 4,653,818 | 3,990,382 | 2,194,442 | 252,194 |
| 3 | 6 | 8 | SNEB | 14,862,278 | 3,114,354 | 2,428,429 | 9,319,495 | 5,063,329 | 4,256,166 | 2,334,234 | 292,153 |
| 3 | 7 | 11 | SNEB | 14,671,705 | 2,896,942 | 2,470,061 | 9,304,702 | 5,251,104 | 4,053,598 | 2,204,508 | 290,920 |
| 3 | 8 | 12 | SNEB | 14,072,947 | 2,763,059 | 2,469,894 | 8,839,994 | 5,237,608 | 3,602,386 | 2,076,201 | 243,862 |
|  |  |  | **Average** | 13,664,739 | 2,819,782 | 2,305,524 | 8,539,432 | 4,717,477 | 3,821,955 | 2,032,130 | 265,322 |
