# Supplementary material for: RNA-seq analysis of differential gene expression in liver from lactating dairy cows divergent in negative energy balance
Source: BMC Genomics. 2012 May 20;13:193. doi: 10.1186/1471-2164-13-193 (PMC3465249; doi:10.1186/1471-2164-13-193)
Supplement: Additional file 4 — Graph of effect of SNEB compared to MNEB on blood concentrations of major NEB associated metabolites 1–14 days post-calving. [file 1471-2164-13-193-S4.pdf]

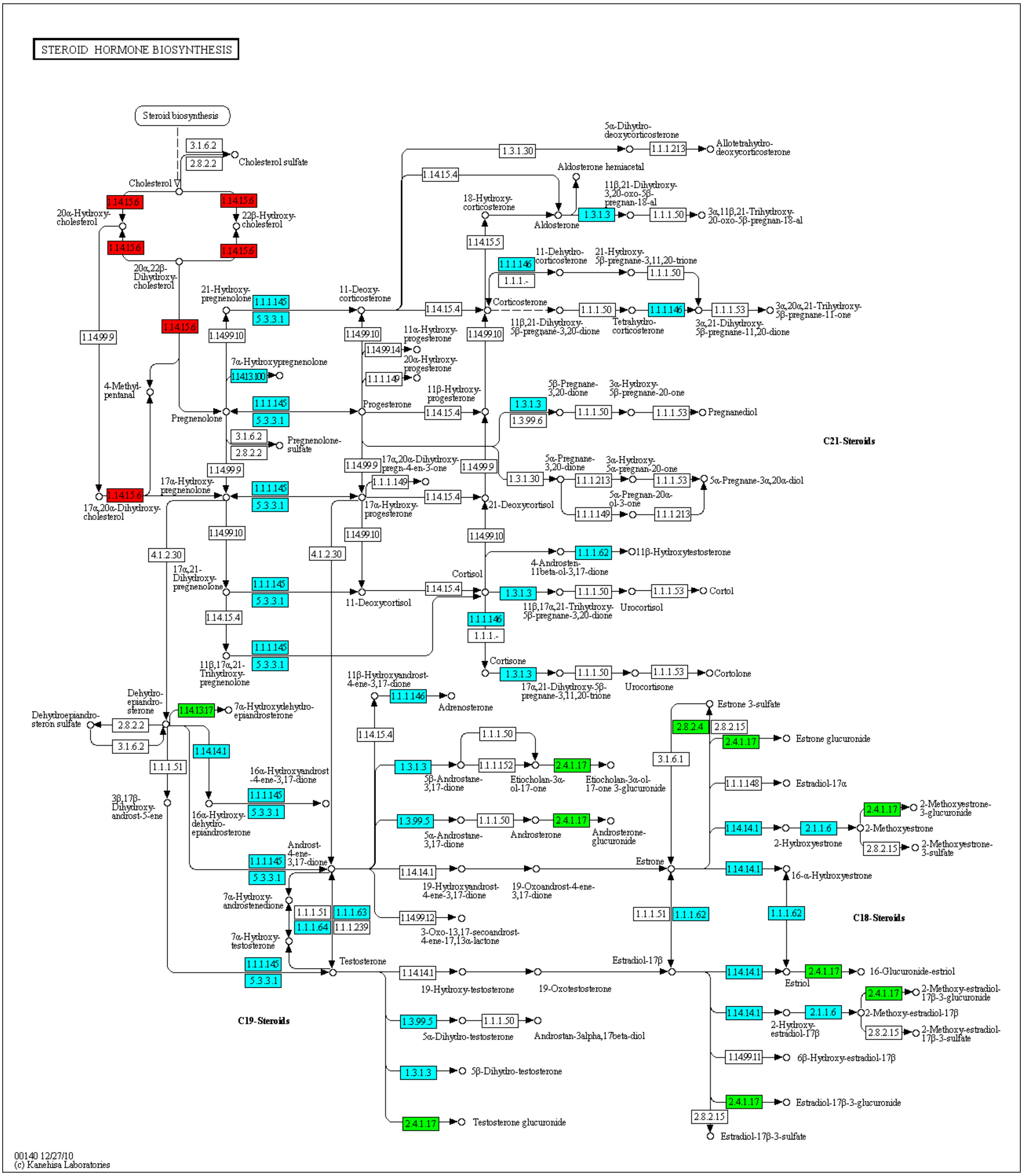

STEROID HORMONE BIOSYNTHESIS
Steroid biosynthesis
Cholesterol
3.1.6.2
2.8.2.2
Cholesterol sulfate
1.14.15.6
20α-Hydroxy-cholesterol
22β-Hydroxy-cholesterol
20α,22β-Dihydroxy-cholesterol
1.14.99.9
4-Methyl-pentanal
Pregnenolone
Pregnenolone-sulfate
21-Hydroxy-pregnenolone
1.1.1.145
5.3.3.1
1.14.99.10
7α-Hydroxypregnenolone
1.14.13.100
17α-Hydroxy-pregnenolone
17α,20α-Dihydroxy-cholesterol
4.1.2.30
17α,21-Dihydroxy-pregnenolone
1.14.15.4
11β,17α,21-Trihydroxy-pregnenolone
11-Deoxy-corticosterone
11α-Hydroxy-progesterone
1.14.99.14
20α-Hydroxy-progesterone
1.1.1.149
11β-Hydroxy-progesterone
Progesterone
17α,20α-Dihydroxy-pregn-4-en-3-one
17α-Hydroxy-progesterone
11-Deoxycortisol
21-Deoxycortisol
Cortisol
1.3.1.30
5α-Dihydro-deoxycorticosterone
1.1.1.213
Allotetrahydro-deoxycorticosterone
Aldosterone hemiacetal
18-Hydroxy-corticosterone
Aldosterone
1.3.1.3
11β,21-Dihydroxy-3,20-oxo-5β-pregnan-18-al
1.1.1.50
3α,11β,21-Trihydroxy-20-oxo-5β-pregnan-18-al
1.14.15.5
11-Dehydro-corticosterone
21-Hydroxy-5β-pregnane-3,11,20-trione
1.1.1.146
1.1.1.-
Corticosterone
11β,21-Dihydroxy-5β-pregnane-3,20-dione
Tetrahydro-corticosterone
3α,21-Dihydroxy-5β-pregnane-11,20-dione
1.1.1.53
3α,20α,21-Trihydroxy-5β-pregnane-11-one
5β-Pregnane-3,20-dione
3α-Hydroxy-5β-pregnane-20-one
Pregnanediol
1.3.99.6
5α-Pregnane-3,20-dione
3α-Hydroxy-5α-pregnan-20-one
5α-Pregnane-3α,20α-diol
5α-Pregnan-20α-ol-3-one
C21-Steroids
1.1.1.62
11β-Hydroxytestosterone
4-Androsten-11beta-ol-3,17-dione
11β,17α,21-Trihydroxy-5β-pregnane-3,20-dione
Urocortisol
Cortol
Cortisone
17α,21-Dihydroxy-5β-pregnane-3,11,20-trione
Urocortisone
Cortolone
11β-Hydroxyandrost-4-ene-3,17-dione
1.1.1.146
Adrenosterone
Dehydro-epiandro-sterone
1.14.13.17
7α-Hydroxydehydro-epiandrosterone
Dehydroepiandro-steron sulfate
2.8.2.2
3.1.6.2
1.1.1.51
3β,17β-Dihydroxy-androst-5-ene
1.14.14.1
16α-Hydroxyandrost-4-ene-3,17-dione
16α-Hydroxy-dehydro-epiandrosterone
Androst-4-ene-3,17-dione
7α-Hydroxy-androstenedione
1.1.1.63
1.1.1.64
1.1.1.239
7α-Hydroxy-testosterone
Testosterone
C19-Steroids
5β-Androstane-3,17-dione
1.1.1.152
Etiocholan-3α-ol-17-one
2.4.1.17
Etiocholan-3α-ol-17-one 3-glucuronide
1.3.99.5
5α-Androstane-3,17-dione
Androsterone
Androsterone-glucuronide
19-Hydroxyandrost-4-ene-3,17-dione
19-Oxoandrost-4-ene-3,17-dione
1.14.99.12
3-Oxo-13,17-secoandrost-4-ene-17,13α-lactone
19-Hydroxy-testosterone
19-Oxotestosterone
5α-Dihydro-testosterone
Androstan-3alpha,17beta-diol
5β-Dihydro-testosterone
Testosterone glucuronide
Estrone 3-sulfate
2.8.2.4
2.8.2.15
Estrone glucuronide
3.1.6.1
1.1.1.148
Estradiol-17α
2-Methoxyestrone-3-glucuronide
2.1.1.6
2-Methoxyestrone
2-Hydroxyestrone
2-Methoxyestrone-3-sulfate
Estrone
16-α-Hydroxyestrone
C18-Steroids
Estradiol-17β
Estriol
16-Glucuronide-estriol
2-Methoxy-estradiol-17β-3-glucuronide
2-Methoxy-estradiol-17β
2-Hydroxy-estradiol-17β
2-Methoxy-estradiol-17β-3-sulfate
1.14.99.11
6β-Hydroxy-estradiol-17β
Estradiol-17β-3-glucuronide
Estradiol-17β-3-sulfate
00140 12/27/10
(c) Kanehisa Laboratories

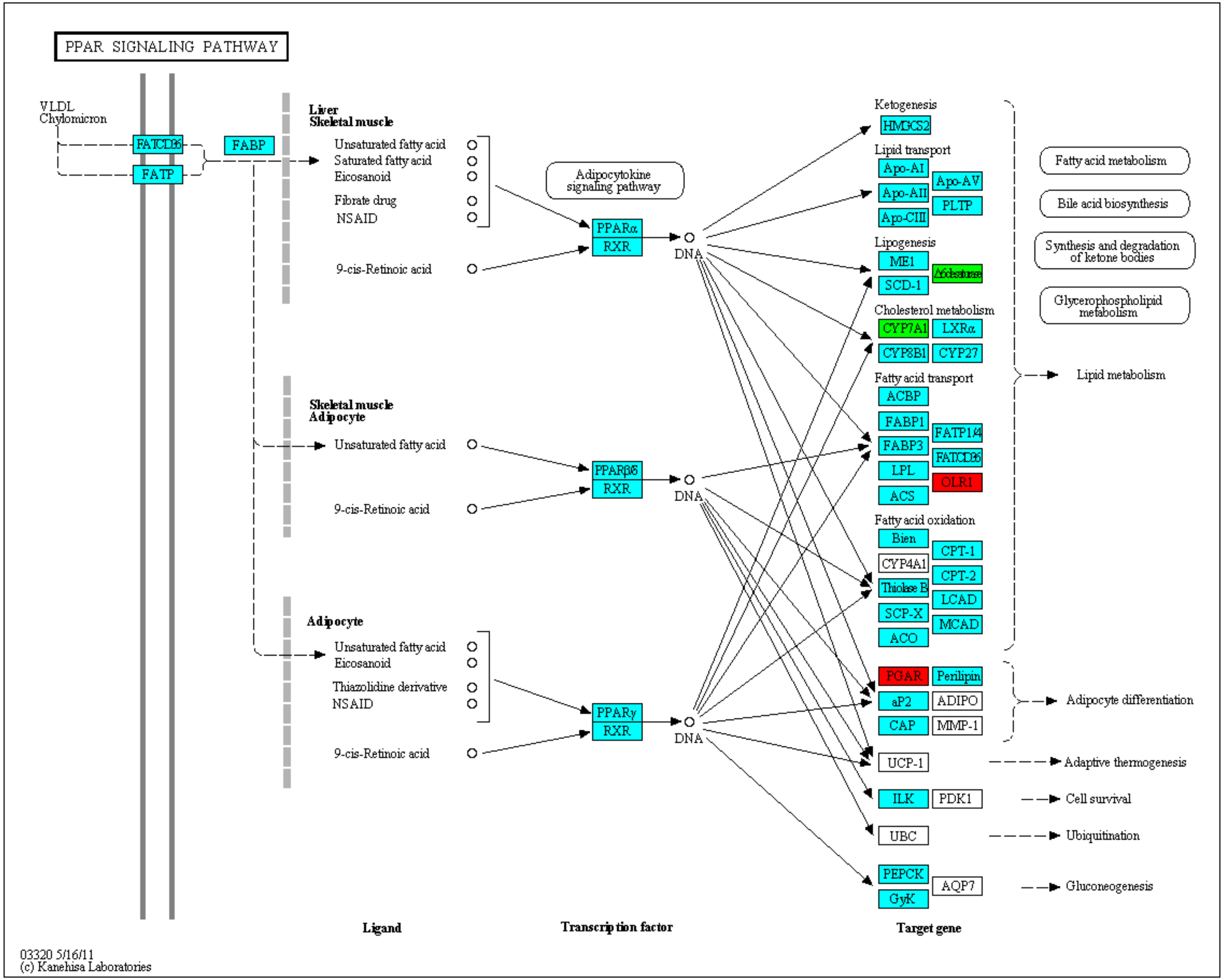
PPAR SIGNALING PATHWAY
VLDL
Chylomicron
FATCD36
FATP
FABP
Liver
Skeletal muscle
Unsaturated fatty acid
Saturated fatty acid
Eicosanoid
Fibrate drug
NSAID
9-cis-Retinoic acid
Adipocytokine signaling pathway
PPARα
RXR
DNA
Skeletal muscle
Adipocyte
Unsaturated fatty acid
9-cis-Retinoic acid
PPARβδ
RXR
DNA
Adipocyte
Unsaturated fatty acid
Eicosanoid
Thiazolidine derivative
NSAID
9-cis-Retinoic acid
PPARγ
RXR
DNA
Ketogenesis
HMGCS2
Lipid transport
Apo-AI
Apo-AII
Apo-CIII
Apo-AV
PLTP
Lipogenesis
ME1
SCD-1
Cholesterol metabolism
CYP7A1
LXRα
CYP8B1
CYP27
Fatty acid transport
ACBP
FABP1
FABP3
LPL
ACS
FATP1/4
FATCD36
OLR1
Fatty acid oxidation
Bien
CYP4A1
Thiolase B
SCP-X
ACO
CPT-1
CPT-2
LCAD
MCAD
PGAR
Perilipin
aP2
ADIPO
CAP
MMP-1
UCP-1
ILK
PDK1
UBC
PEPCK
GyK
AQP7
Fatty acid metabolism
Bile acid biosynthesis
Synthesis and degradation of ketone bodies
Glycerophospholipid metabolism
Lipid metabolism
Adipocyte differentiation
Adaptive thermogenesis
Cell survival
Ubiquitination
Gluconeogenesis
Ligand
Transcription factor
Target gene
03320 5/16/11
(c) Kanehisa Laboratories

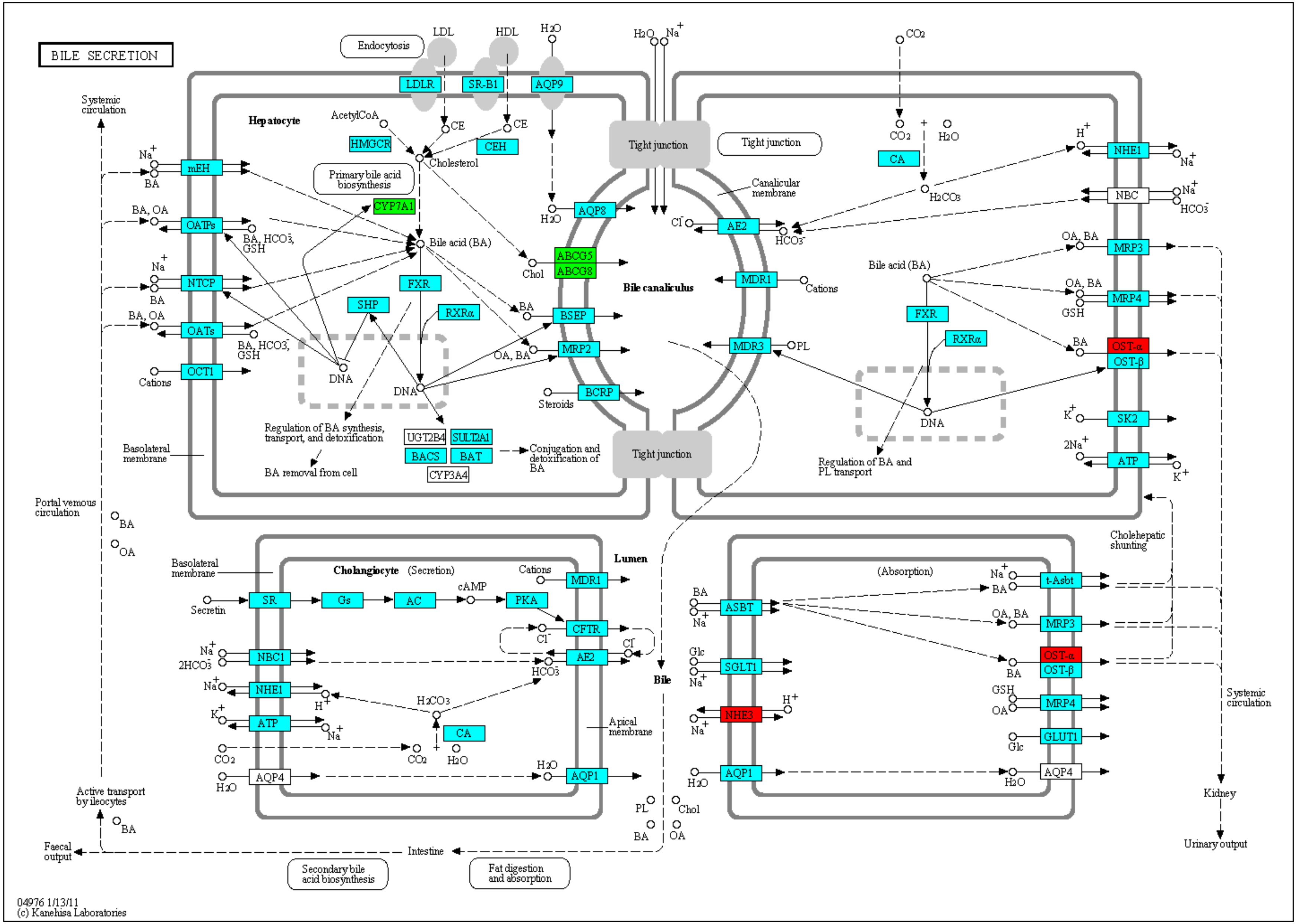
BILE SECRETION
Systemic circulation
Endocytosis
LDL
HDL
LDLR
SR-B1
AQP9
Hepatocyte
AcetylCoA
HMGCR
CE
CEH
Cholesterol
Primary bile acid biosynthesis
CYP7A1
Bile acid (BA)
FXR
SHP
RXRα
DNA
Regulation of BA synthesis, transport, and detoxification
BA removal from cell
UGT2B4
SULT2A1
BACS
BAT
CYP3A4
Conjugation and detoxification of BA
mEH
OATPs
NTCP
OATs
OCT1
Cations
Basolateral membrane
Portal venous circulation
ABCG5
ABCG8
Chol
AQP8
BSEP
MRP2
BCRP
Steroids
Tight junction
Bile canaliculus
Canalicular membrane
AE2
MDR1
MDR3
PL
CA
NHE1
NBC
MRP3
MRP4
OST-α
OST-β
SK2
ATP
Regulation of BA and PL transport
Cholehepatic shunting
Cholangiocyte (Secretion)
Lumen
Secretin
SR
Gs
AC
cAMP
PKA
CFTR
NBC1
NHE1
AQP4
AQP1
Apical membrane
Bile
(Absorption)
ASBT
SGLT1
NHE3
t-Asbt
GLUT1
Systemic circulation
Kidney
Urinary output
Active transport by ileocytes
Faecal output
Intestine
Secondary bile acid biosynthesis
Fat digestion and absorption
04976 1/13/11
(c) Kanehisa Laboratories

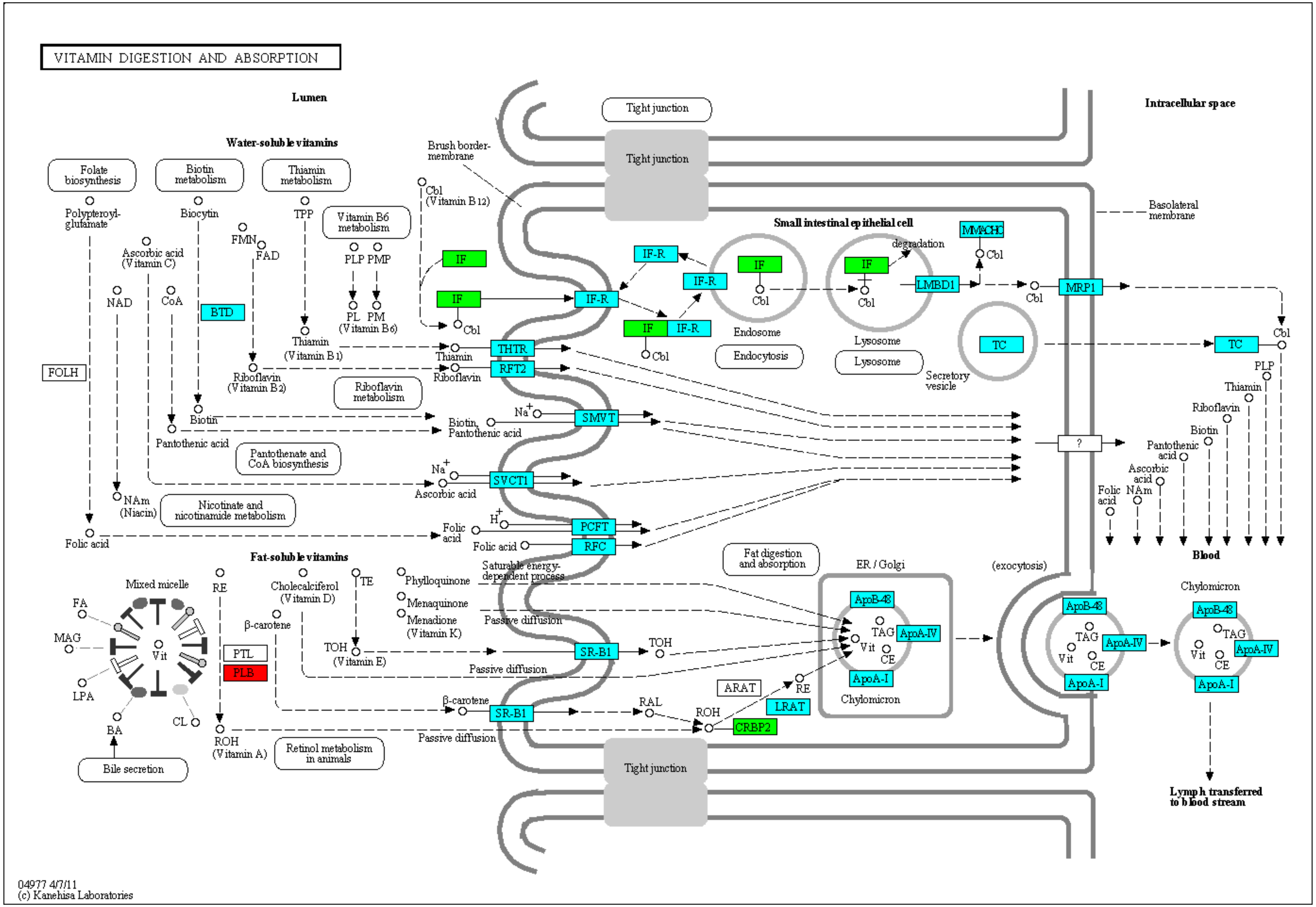
VITAMIN DIGESTION AND ABSORPTION
Lumen
Intracellular space
Water-soluble vitamins
Folate biosynthesis
Biotin metabolism
Thiamin metabolism
Vitamin B6 metabolism
Brush border-membrane
Tight junction
Polypteroyl-glutamate
Biocytin
TPP
FMN
FAD
PLP
PMP
Ascorbic acid (Vitamin C)
NAD
CoA
BTD
PL
PM
(Vitamin B6)
Thiamin (Vitamin B1)
Riboflavin (Vitamin B2)
Riboflavin metabolism
FOLH
Biotin
Pantothenic acid
Pantothenate and CoA biosynthesis
NAm (Niacin)
Nicotinate and nicotinamide metabolism
Folic acid
Cbl (Vitamin B12)
IF
IF-R
Cbl
Thiamin
THTR
Riboflavin
RFT2
Biotin, Pantothenic acid
Na+
SMVT
Ascorbic acid
SVCT1
H+
Folic acid
PCFT
RFC
Small intestinal epithelial cell
Endosome
Endocytosis
degradation
Lysosome
LMBD1
MMACHC
MRP1
TC
Secretory vesicle
Basolateral membrane
PLP
Thiamin
Riboflavin
Biotin
Pantothenic acid
Ascorbic acid
Folic acid
NAm
?
Blood
Fat-soluble vitamins
Mixed micelle
FA
MAG
LPA
BA
CL
Vit
Bile secretion
RE
PTL
PLB
ROH (Vitamin A)
Retinol metabolism in animals
Cholecalciferol (Vitamin D)
β-carotene
TE
TOH (Vitamin E)
Phylloquinone
Menaquinone
Menadione (Vitamin K)
Saturable energy-dependent process
Passive diffusion
SR-B1
TOH
Fat digestion and absorption
ER / Golgi
ApoB-48
TAG
ApoA-IV
Vit
CE
ApoA-I
Chylomicron
ARAT
RE
LRAT
RAL
ROH
CRBP2
(exocytosis)
Lymph transferred to blood stream
04977 4/7/11
(c) Kanehisa Laboratories

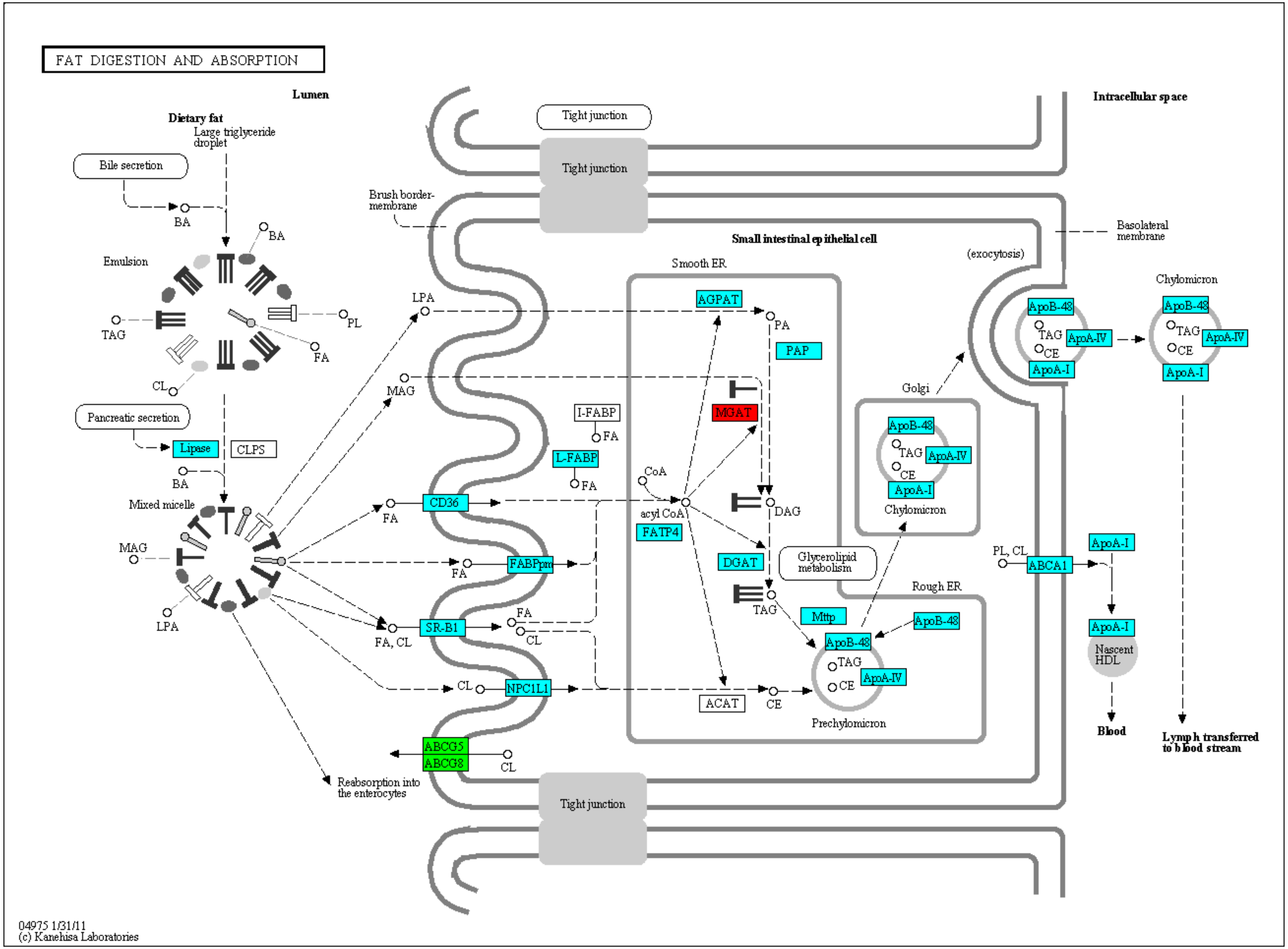
FAT DIGESTION AND ABSORPTION
Lumen
Intracellular space
Dietary fat
Large triglyceride droplet
Bile secretion
BA
Emulsion
TAG
PL
FA
CL
Pancreatic secretion
Lipase
CLPS
Mixed micelle
MAG
LPA
Brush border-membrane
Tight junction
Small intestinal epithelial cell
Smooth ER
AGPAT
PA
PAP
MGAT
I-FABP
L-FABP
CoA
acyl CoA
FATP4
DAG
DGAT
Glycerolipid metabolism
CD36
FABPpm
SR-B1
FA, CL
NPC1L1
ABCG5
ABCG8
ACAT
CE
Mttp
ApoB-48
ApoA-IV
ApoA-I
Prechylomicron
Rough ER
Golgi
Chylomicron
(exocytosis)
Basolateral membrane
PL, CL
ABCA1
Nascent HDL
Blood
Lymph transferred to blood stream
Reabsorption into the enterocytes
04975 1/31/11
(c) Kanehisa Laboratories

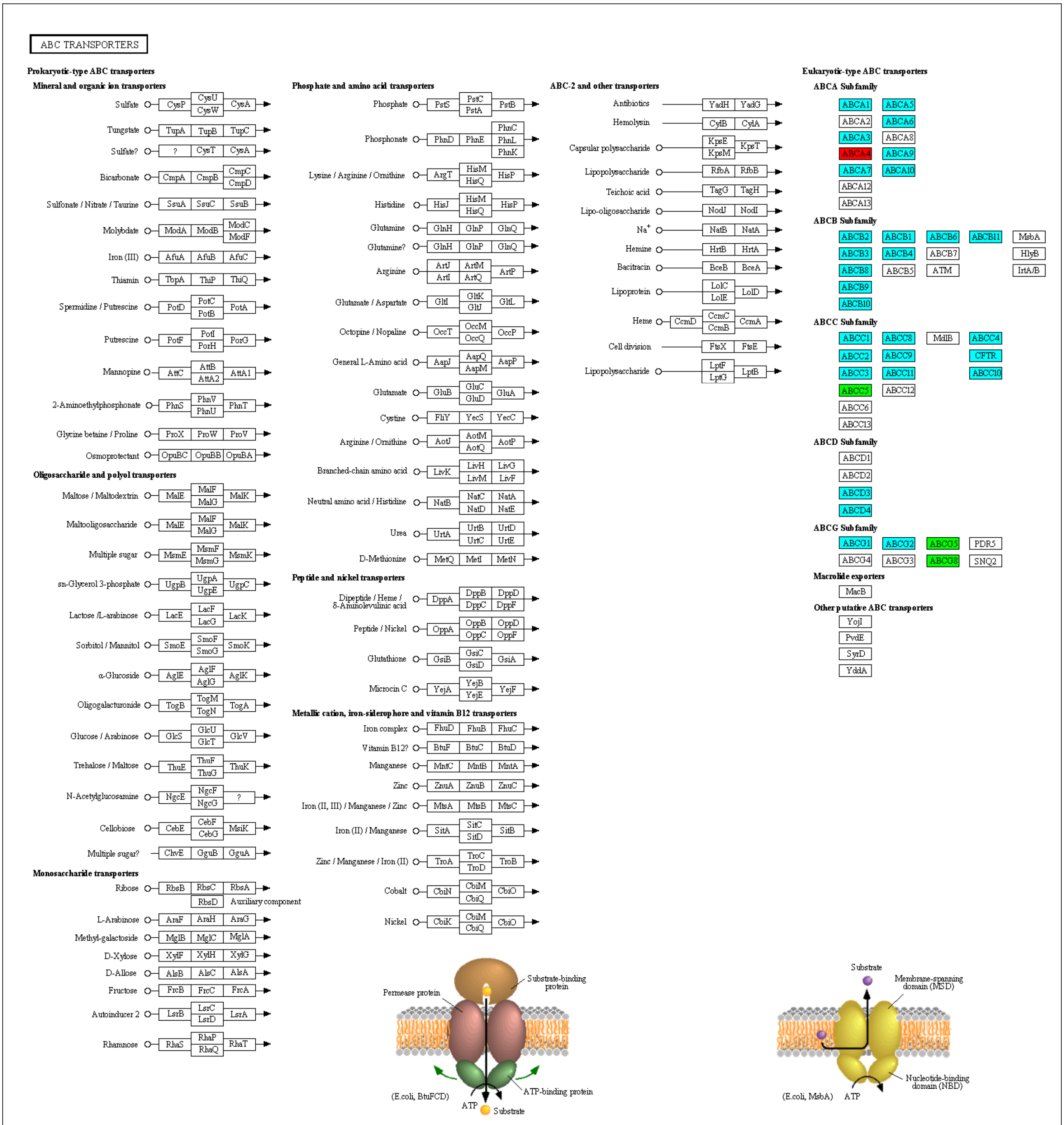

02010 3/25/11
(c) Kanehisa Laboratories

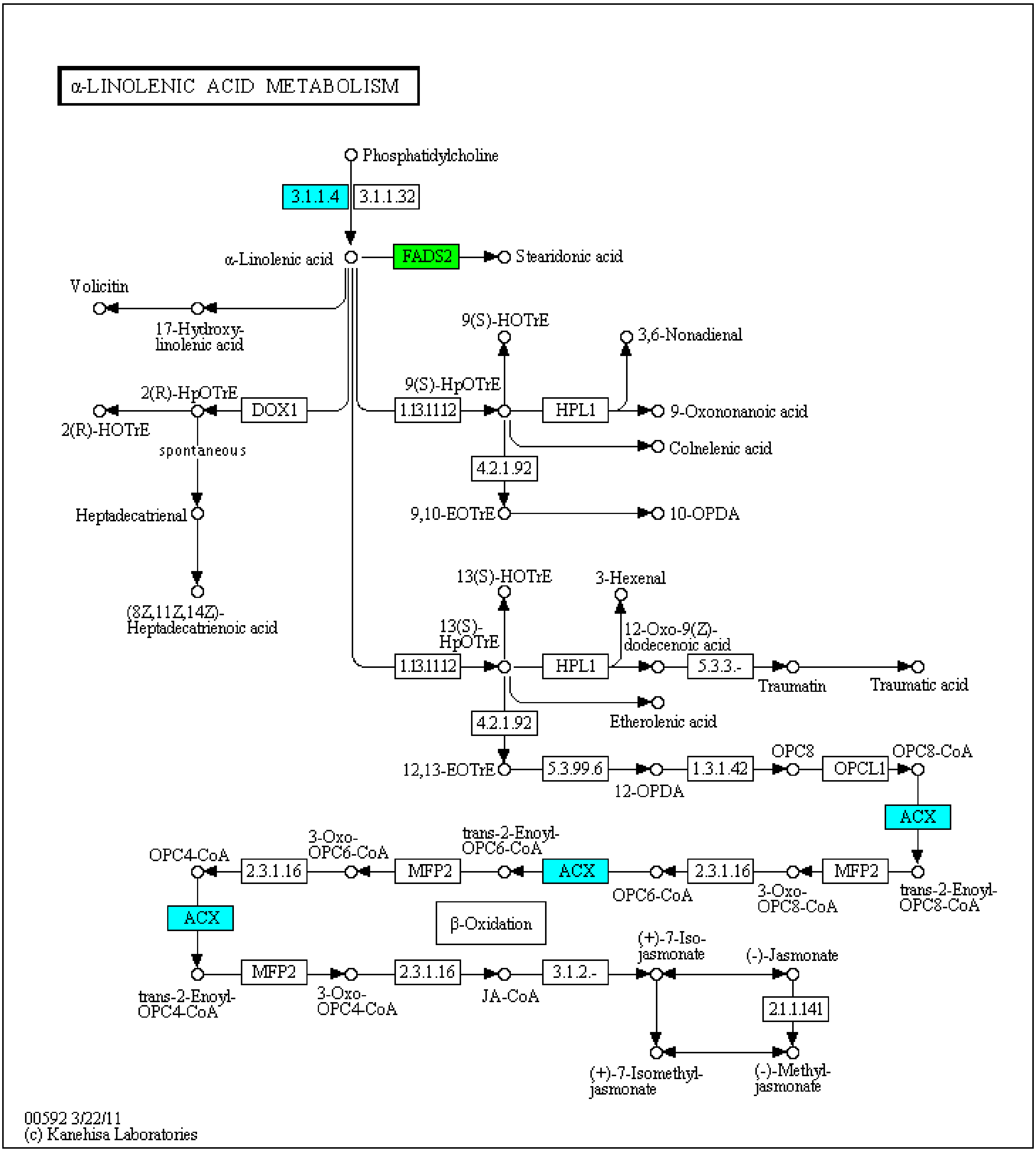

α-LINOLENIC ACID METABOLISM
Phosphatidylcholine
3.1.1.4
3.1.1.32
α-Linolenic acid
FADS2
Stearidonic acid
Volicitin
17-Hydroxy-linolenic acid
2(R)-HpOTrE
DOX1
2(R)-HOTrE
spontaneous
Heptadecatrienal
(8Z,11Z,14Z)-Heptadecatrienoic acid
9(S)-HOTrE
3,6-Nonadienal
9(S)-HpOTrE
1.13.11.12
HPL1
9-Oxononanoic acid
Colnelenic acid
4.2.1.92
9,10-EOTrE
10-OPDA
13(S)-HOTrE
3-Hexenal
13(S)-HpOTrE
12-Oxo-9(Z)-dodecenoic acid
1.13.11.12
HPL1
5.3.3.-
Traumatin
Traumatic acid
Etherolenic acid
4.2.1.92
12,13-EOTrE
5.3.99.6
12-OPDA
1.3.1.42
OPC8
OPCL1
OPC8-CoA
ACX
trans-2-Enoyl-OPC8-CoA
MFP2
3-Oxo-OPC8-CoA
2.3.1.16
OPC6-CoA
ACX
trans-2-Enoyl-OPC6-CoA
MFP2
3-Oxo-OPC6-CoA
2.3.1.16
OPC4-CoA
ACX
β-Oxidation
trans-2-Enoyl-OPC4-CoA
MFP2
3-Oxo-OPC4-CoA
2.3.1.16
JA-CoA
3.1.2.-
(+)-7-Iso-jasmonate
(-)-Jasmonate
2.1.1.141
(+)-7-Isomethyl-jasmonate
(-)-Methyl-jasmonate
00592 3/22/11
(c) Kanehisa Laboratories

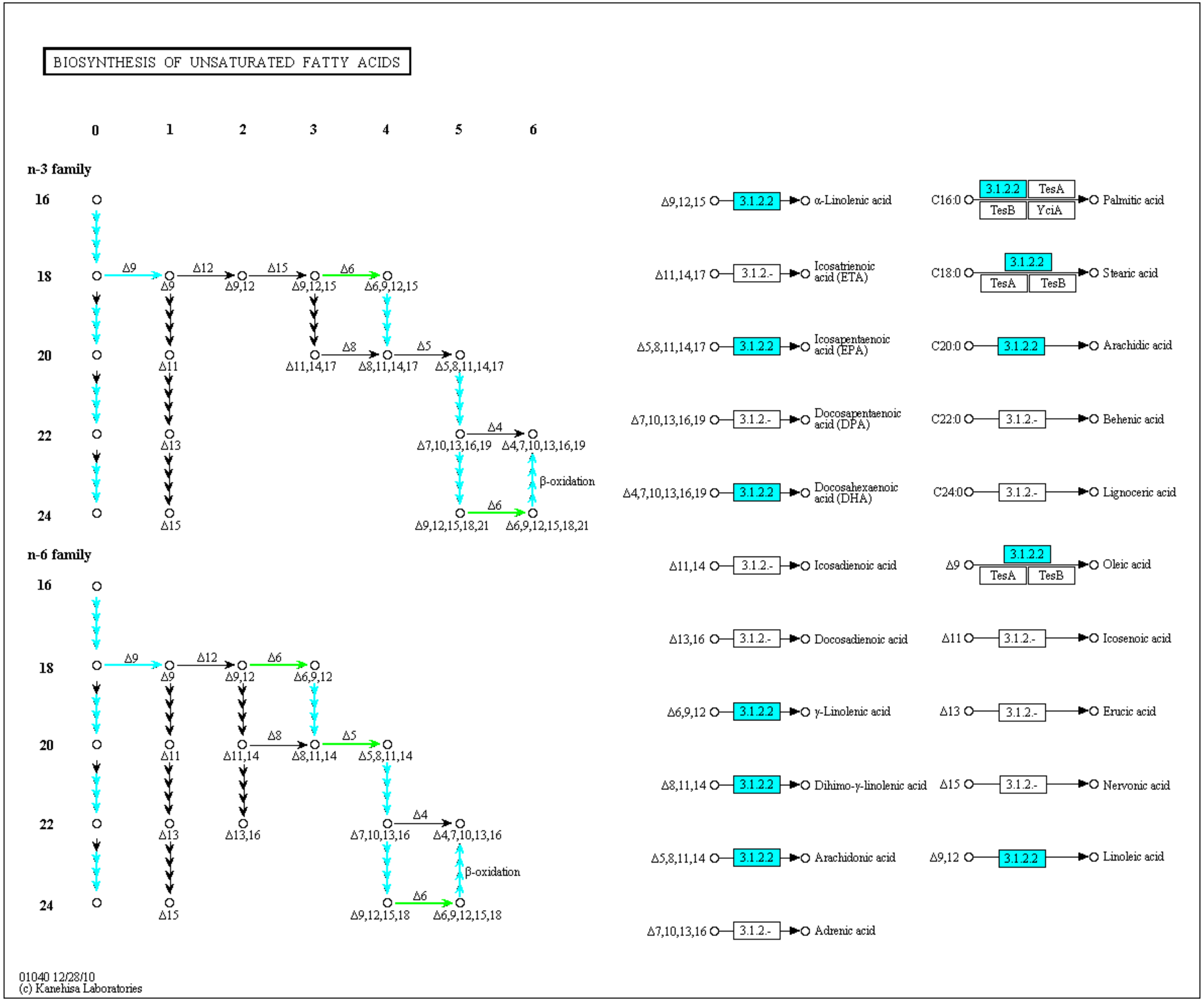

BIOSYNTHESIS OF UNSATURATED FATTY ACIDS
0 1 2 3 4 5 6
n-3 family
16
18
20
22
24
Δ9 Δ12 Δ15 Δ6
Δ9 Δ9,12 Δ9,12,15 Δ6,9,12,15
Δ8 Δ5
Δ11 Δ11,14,17 Δ8,11,14,17 Δ5,8,11,14,17
Δ4
Δ13 Δ7,10,13,16,19 Δ4,7,10,13,16,19
β-oxidation
Δ6
Δ15 Δ9,12,15,18,21 Δ6,9,12,15,18,21
n-6 family
16
18
20
22
24
Δ9 Δ12 Δ6
Δ9 Δ9,12 Δ6,9,12
Δ8 Δ5
Δ11 Δ11,14 Δ8,11,14 Δ5,8,11,14
Δ4
Δ13 Δ13,16 Δ7,10,13,16 Δ4,7,10,13,16
β-oxidation
Δ6
Δ15 Δ9,12,15,18 Δ6,9,12,15,18
Δ9,12,15 3.1.2.2 α-Linolenic acid
Δ11,14,17 3.1.2.- Icosatrienoic acid (ETA)
Δ5,8,11,14,17 3.1.2.2 Icosapentaenoic acid (EPA)
Δ7,10,13,16,19 3.1.2.- Docosapentaenoic acid (DPA)
Δ4,7,10,13,16,19 3.1.2.2 Docosahexaenoic acid (DHA)
Δ11,14 3.1.2.- Icosadienoic acid
Δ13,16 3.1.2.- Docosadienoic acid
Δ6,9,12 3.1.2.2 γ-Linolenic acid
Δ8,11,14 3.1.2.2 Dihimo-γ-linolenic acid
Δ5,8,11,14 3.1.2.2 Arachidonic acid
Δ7,10,13,16 3.1.2.- Adrenic acid
C16:0 3.1.2.2 TesA TesB YciA Palmitic acid
C18:0 3.1.2.2 TesA TesB Stearic acid
C20:0 3.1.2.2 Arachidic acid
C22:0 3.1.2.- Behenic acid
C24:0 3.1.2.- Lignoceric acid
Δ9 3.1.2.2 TesA TesB Oleic acid
Δ11 3.1.2.- Icosenoic acid
Δ13 3.1.2.- Erucic acid
Δ15 3.1.2.- Nervonic acid
Δ9,12 3.1.2.2 Linoleic acid
01040 12/28/10
(c) Kanehisa Laboratories

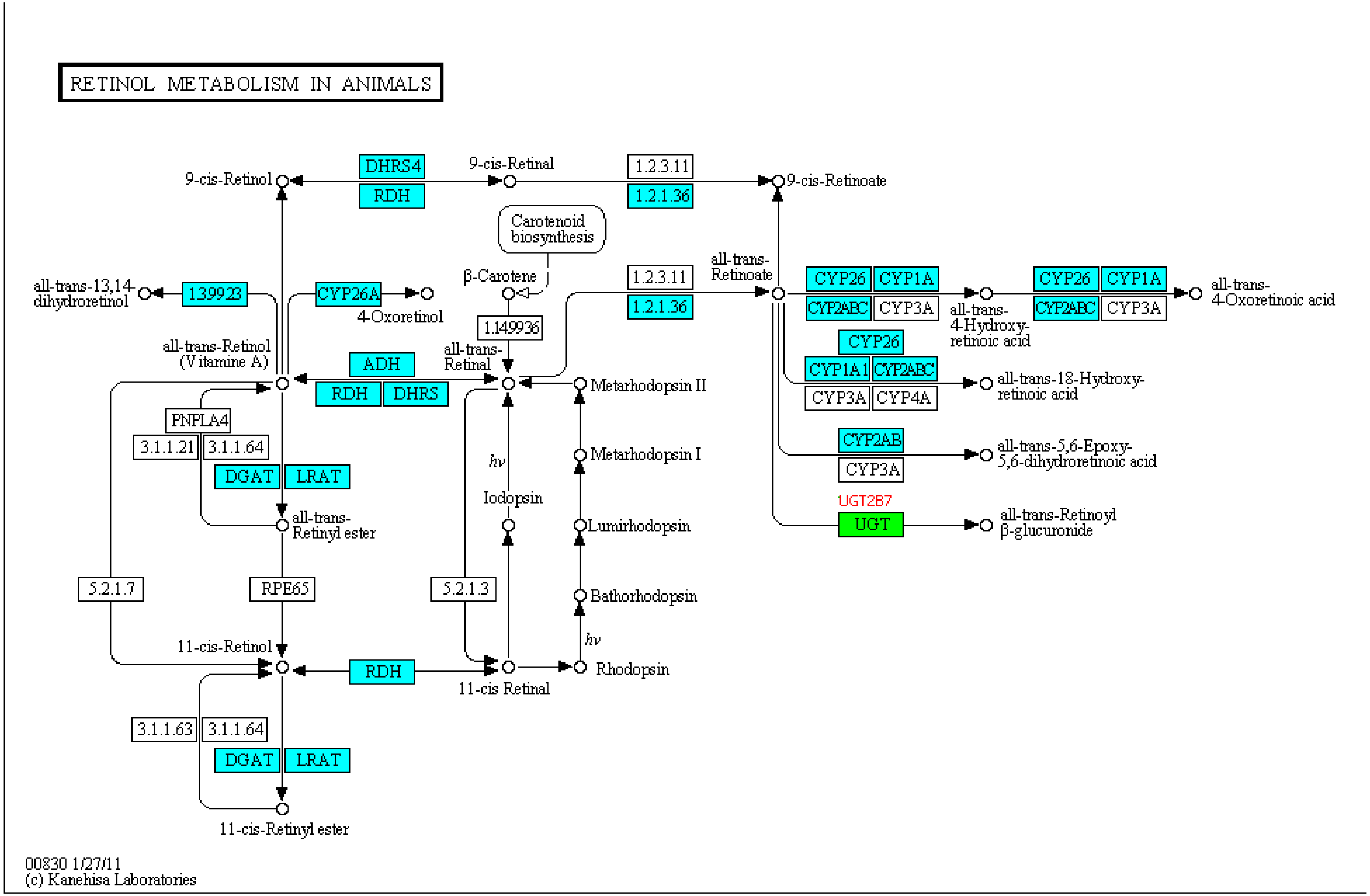
RETINOL METABOLISM IN ANIMALS
9-cis-Retinol
DHRS4
RDH
9-cis-Retinal
1.2.3.11
1.2.1.36
9-cis-Retinoate
Carotenoid biosynthesis
β-Carotene
all-trans-13,14-dihydroretinol
139923
CYP26A
4-Oxoretinol
1.2.3.11
1.2.1.36
all-trans-Retinoate
CYP26
CYP1A
CYP2ABC
CYP3A
all-trans-4-Hydroxy-retinoic acid
CYP26
CYP1A
CYP2ABC
CYP3A
all-trans-4-Oxoretinoic acid
1.14.99.36
all-trans-Retinol (Vitamine A)
ADH
RDH
DHRS
all-trans-Retinal
Metarhodopsin II
CYP26
CYP1A1
CYP2ABC
CYP3A
CYP4A
all-trans-18-Hydroxy-retinoic acid
PNPLA4
3.1.1.21
3.1.1.64
DGAT
LRAT
hv
Metarhodopsin I
CYP2AB
CYP3A
all-trans-5,6-Epoxy-5,6-dihydroretinoic acid
Iodopsin
UGT2B7
UGT
all-trans-Retinoyl β-glucuronide
all-trans-Retinyl ester
Lumirhodopsin
5.2.1.7
RPE65
5.2.1.3
Bathorhodopsin
hv
11-cis-Retinol
RDH
Rhodopsin
11-cis Retinal
3.1.1.63
3.1.1.64
DGAT
LRAT
11-cis-Retinyl ester
00830 1/27/11
(c) Kanehisa Laboratories
