# Supplementary material for: RNA-seq analysis of differential gene expression in liver from lactating dairy cows divergent in negative energy balance
Source: BMC Genomics. 2012 May 20;13:193. doi: 10.1186/1471-2164-13-193 (PMC3465249; doi:10.1186/1471-2164-13-193)
Supplement: Additional file 5 — Maps of KEGG pathways (associated with ≥2 fold (FDR 0.1%) SDE genes) overrepresented in SNEB animals. [file 1471-2164-13-193-S5.pdf]

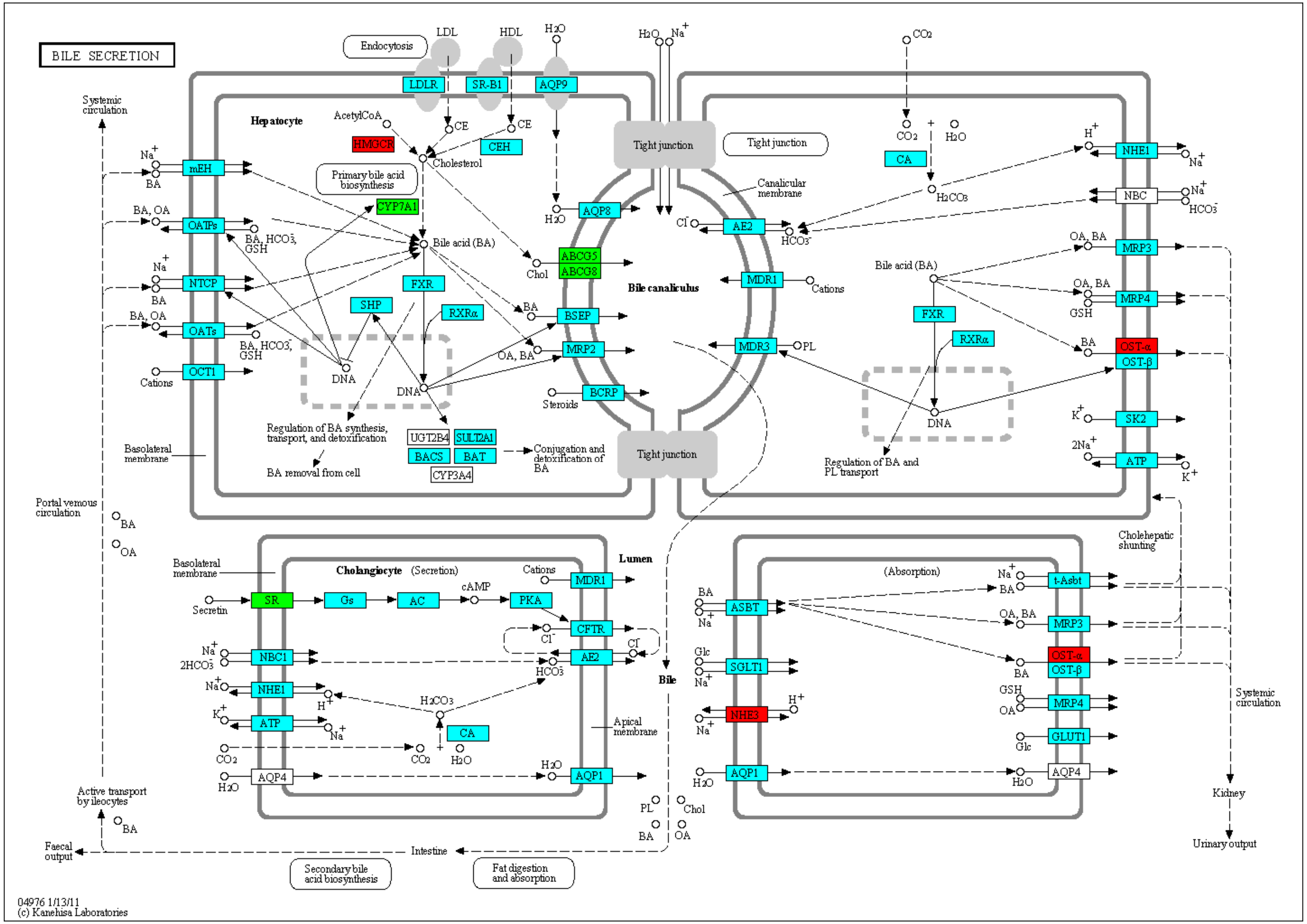
BILE SECRETION
Endocytosis
LDL
HDL
H2O
LDLR
SR-B1
AQP9
Systemic circulation
Hepatocyte
AcetylCoA
HMGCR
CE
CEH
Cholesterol
Primary bile acid biosynthesis
CYP7A1
Na+
BA
mEH
BA, OA
OATPs
BA, HCO3-, GSH
Bile acid (BA)
NTCP
FXR
SHP
RXRα
OATs
OCT1
Cations
DNA
Regulation of BA synthesis, transport, and detoxification
BA removal from cell
UGT2B4
SULT2A1
BACS
BAT
CYP3A4
Conjugation and detoxification of BA
Basolateral membrane
Portal venous circulation
AQP8
ABCG5
ABCG8
Chol
BSEP
MRP2
OA, BA
BCRP
Steroids
Tight junction
Canalicular membrane
Bile canaliculus
Cl-
AE2
HCO3-
MDR1
MDR3
PL
CO2
CA
H2CO3
H+
NHE1
NBC
MRP3
MRP4
GSH
OST-α
OST-β
K+
SK2
2Na+
ATP
Regulation of BA and PL transport
Cholehepatic shunting
Cholangiocyte (Secretion)
Lumen
Secretin
SR
Gs
AC
cAMP
PKA
CFTR
NBC1
2HCO3-
AQP4
AQP1
Bile
Apical membrane
(Absorption)
t-Asbt
ASBT
Glc
SGLT1
NHE3
GLUT1
Systemic circulation
Kidney
Urinary output
Active transport by ileocytes
Faecal output
Intestine
Secondary bile acid biosynthesis
Fat digestion and absorption
04976 1/13/11
(c) Kanehisa Laboratories

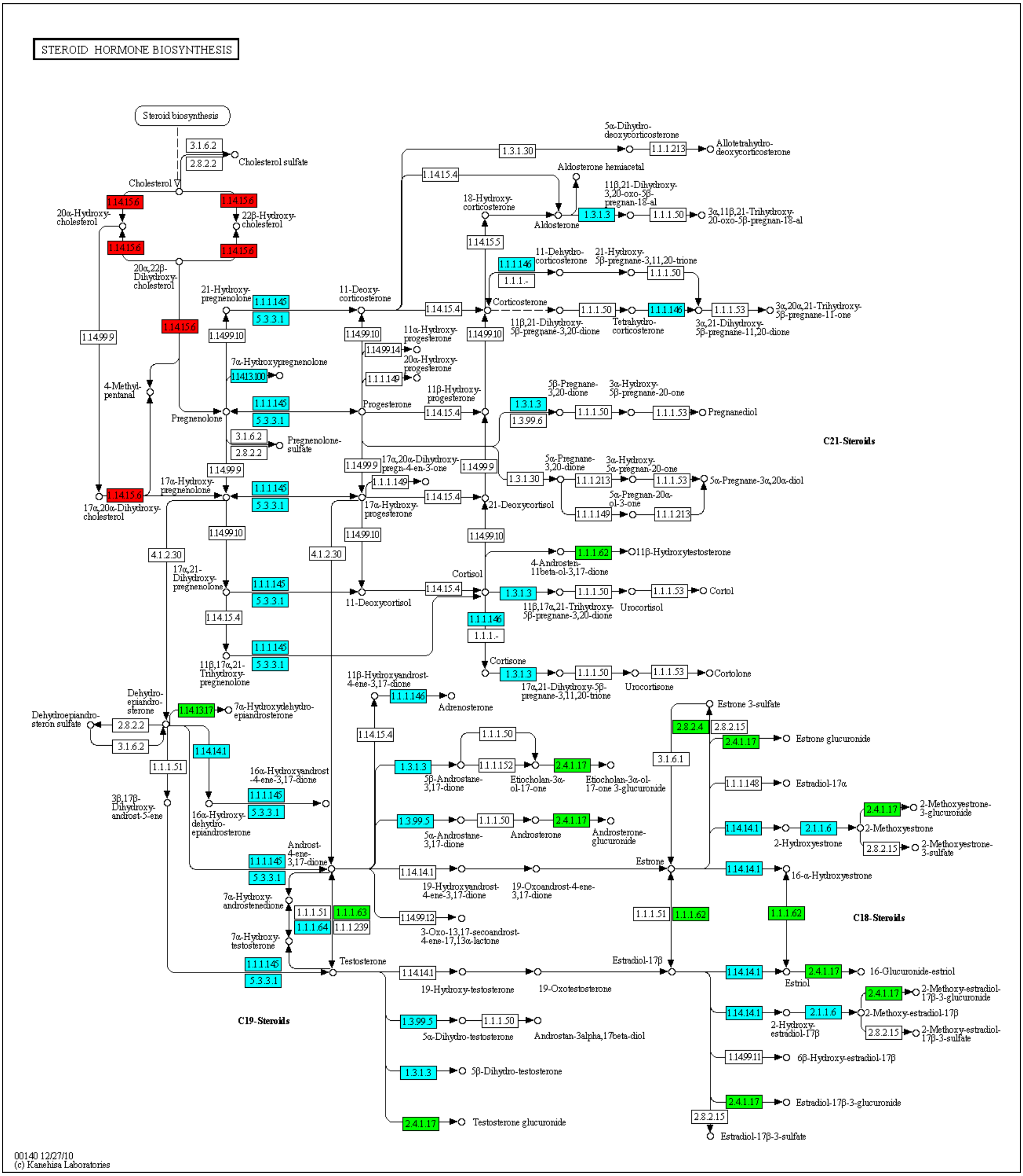
STEROID HORMONE BIOSYNTHESIS
Steroid biosynthesis
3.1.6.2
2.8.2.2
Cholesterol sulfate
Cholesterol
1.14.15.6
20α-Hydroxy-cholesterol
22β-Hydroxy-cholesterol
20α,22β-Dihydroxy-cholesterol
1.14.99.9
4-Methyl-pentanal
Pregnenolone
21-Hydroxy-pregnenolone
1.1.1.145
5.3.3.1
1.14.99.10
7α-Hydroxypregnenolone
1.14.13.100
Pregnenolone-sulfate
17α-Hydroxy-pregnenolone
17α,20α-Dihydroxy-cholesterol
4.1.2.30
17α,21-Dihydroxy-pregnenolone
1.14.15.4
11β,17α,21-Trihydroxy-pregnenolone
11-Deoxy-corticosterone
1.3.1.30
5α-Dihydro-deoxycorticosterone
1.1.1.213
Allotetrahydro-deoxycorticosterone
Aldosterone hemiacetal
18-Hydroxy-corticosterone
Aldosterone
1.3.1.3
11β,21-Dihydroxy-3,20-oxo-5β-pregnan-18-al
1.1.1.50
3α,11β,21-Trihydroxy-20-oxo-5β-pregnan-18-al
1.14.15.5
1.1.1.146
1.1.1.-
11-Dehydro-corticosterone
21-Hydroxy-5β-pregnane-3,11,20-trione
Corticosterone
11β,21-Dihydroxy-5β-pregnane-3,20-dione
Tetrahydro-corticosterone
3α,21-Dihydroxy-5β-pregnane-11,20-dione
1.1.1.53
3α,20α,21-Trihydroxy-5β-pregnane-11-one
1.14.99.14
11α-Hydroxy-progesterone
1.1.1.149
20α-Hydroxy-progesterone
11β-Hydroxy-progesterone
Progesterone
1.3.99.6
5β-Pregnane-3,20-dione
3α-Hydroxy-5β-pregnan-20-one
Pregnanediol
C21-Steroids
17α,20α-Dihydroxy-pregn-4-en-3-one
5α-Pregnane-3,20-dione
3α-Hydroxy-5α-pregnan-20-one
5α-Pregnane-3α,20α-diol
5α-Pregnan-20α-ol-3-one
17α-Hydroxy-progesterone
21-Deoxycortisol
4-Androsten-11beta-ol-3,17-dione
1.1.1.62
11β-Hydroxytestosterone
Cortisol
11-Deoxycortisol
11β,17α,21-Trihydroxy-5β-pregnane-3,20-dione
Urocortisol
Cortol
Cortisone
17α,21-Dihydroxy-5β-pregnane-3,11,20-trione
Urocortisone
Cortolone
11β-Hydroxyandrost-4-ene-3,17-dione
Adrenosterone
Dehydro-epiandro-sterone
1.14.13.17
7α-Hydroxydehydro-epiandrosterone
Dehydroepiandro-steron sulfate
1.1.1.51
1.14.14.1
3β,17β-Dihydroxy-androst-5-ene
16α-Hydroxyandrost-4-ene-3,17-dione
16α-Hydroxy-dehydro-epiandrosterone
Androst-4-ene-3,17-dione
7α-Hydroxy-androstenedione
1.1.1.63
1.1.1.64
1.1.1.239
7α-Hydroxy-testosterone
Testosterone
C19-Steroids
1.1.1.152
2.4.1.17
5β-Androstane-3,17-dione
Etiocholan-3α-ol-17-one
Etiocholan-3α-ol-17-one 3-glucuronide
1.3.99.5
5α-Androstane-3,17-dione
Androsterone
Androsterone-glucuronide
19-Hydroxyandrost-4-ene-3,17-dione
19-Oxoandrost-4-ene-3,17-dione
1.14.99.12
3-Oxo-13,17-secoandrost-4-ene-17,13α-lactone
19-Hydroxy-testosterone
19-Oxotestosterone
5α-Dihydro-testosterone
Androstan-3alpha,17beta-diol
5β-Dihydro-testosterone
Testosterone glucuronide
Estrone 3-sulfate
2.8.2.4
2.8.2.15
3.1.6.1
Estrone glucuronide
1.1.1.148
Estradiol-17α
2-Hydroxyestrone
2.1.1.6
2-Methoxyestrone
2-Methoxyestrone-3-glucuronide
2-Methoxyestrone-3-sulfate
Estrone
16-α-Hydroxyestrone
C18-Steroids
Estradiol-17β
Estriol
16-Glucuronide-estriol
2-Hydroxy-estradiol-17β
2-Methoxy-estradiol-17β
2-Methoxy-estradiol-17β-3-glucuronide
2-Methoxy-estradiol-17β-3-sulfate
1.14.99.11
6β-Hydroxy-estradiol-17β
Estradiol-17β-3-glucuronide
Estradiol-17β-3-sulfate
00140 12/27/10
(c) Kanehisa Laboratories

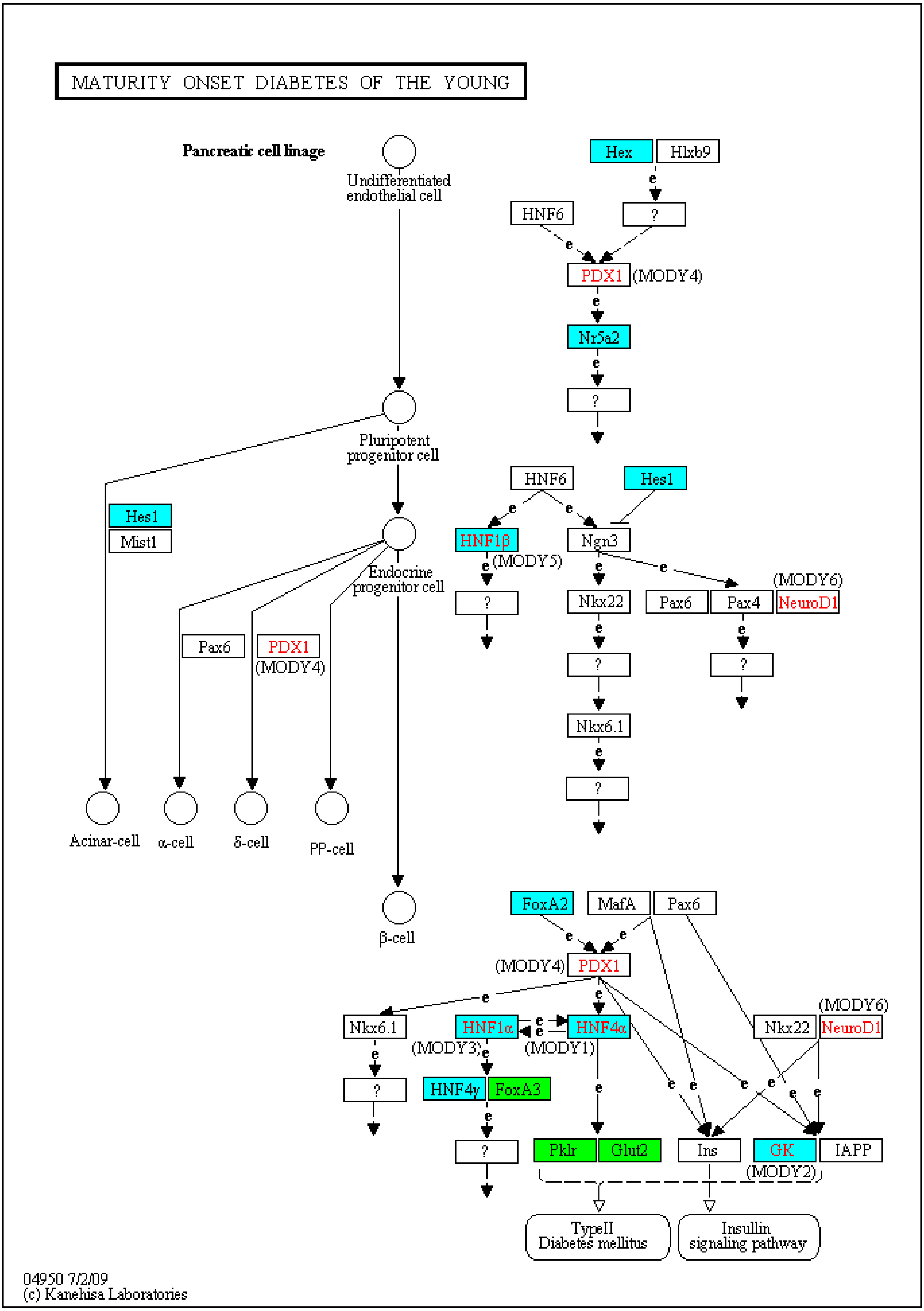
MATURITY ONSET DIABETES OF THE YOUNG
Pancreatic cell linage
Undifferentiated endothelial cell
Pluripotent progenitor cell
Endocrine progenitor cell
Hes1
Mist1
Pax6
PDX1
(MODY4)
Acinar-cell
α-cell
δ-cell
PP-cell
β-cell
Hex
Hlxb9
HNF6
?
PDX1
(MODY4)
Nr5a2
HNF6
Hes1
HNF1β
(MODY5)
Ngn3
Nkx22
Pax6
Pax4
NeuroD1
(MODY6)
Nkx6.1
FoxA2
MafA
Pax6
(MODY4)
PDX1
Nkx6.1
HNF1α
HNF4α
(MODY3)
(MODY1)
Nkx22
NeuroD1
(MODY6)
HNF4γ
FoxA3
Pklr
Glut2
Ins
GK
IAPP
(MODY2)
TypeII Diabetes mellitus
Insullin signaling pathway
e
04950 7/2/09
(c) Kanehisa Laboratories

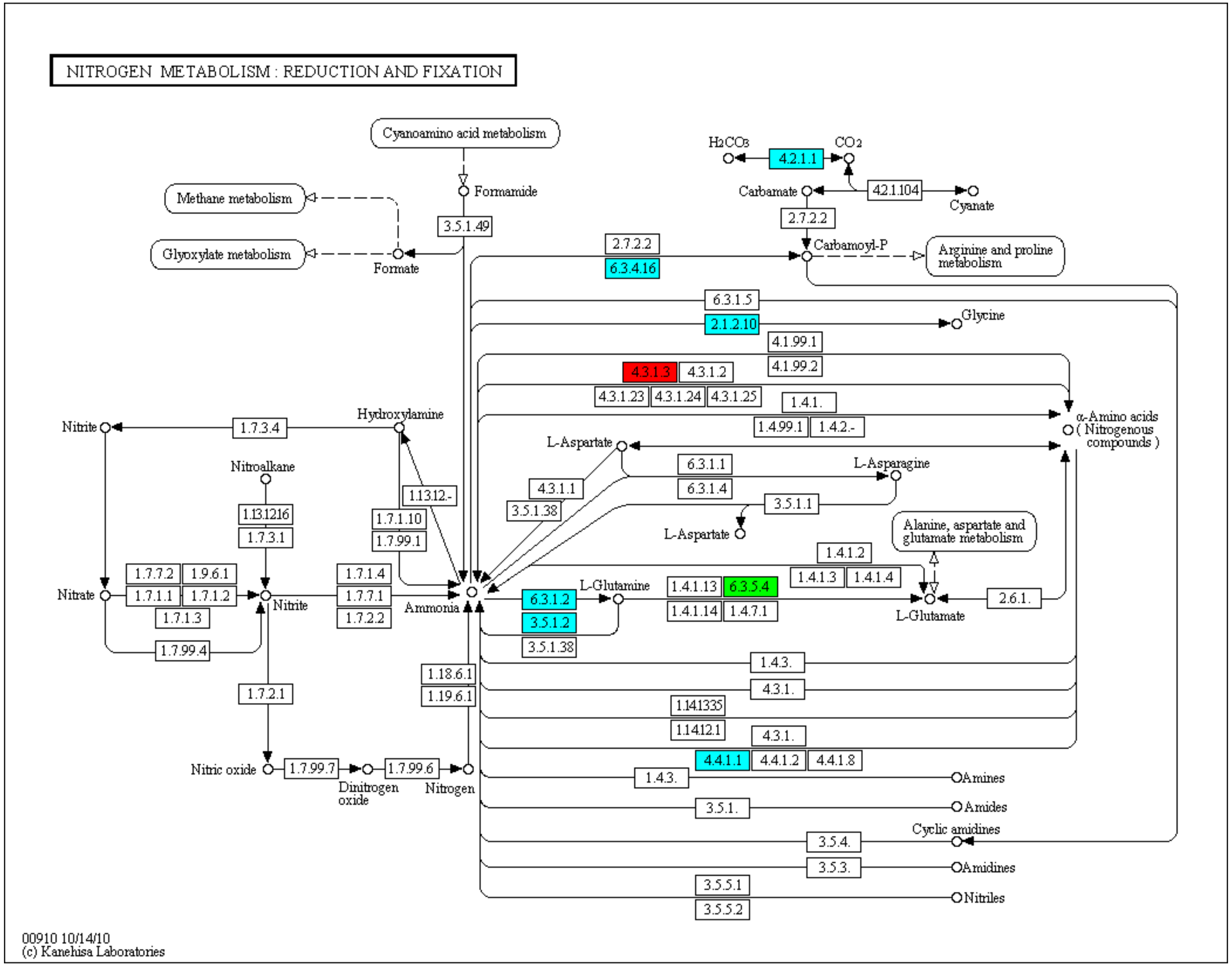
NITROGEN METABOLISM : REDUCTION AND FIXATION
Cyanoamino acid metabolism
Formamide
3.5.1.49
Methane metabolism
Glyoxylate metabolism
Formate
H2CO3
4.2.1.1
CO2
Carbamate
4.2.1.104
Cyanate
2.7.2.2
Carbamoyl-P
Arginine and proline metabolism
2.7.2.2
6.3.4.16
6.3.1.5
2.1.2.10
Glycine
4.1.99.1
4.1.99.2
4.3.1.3
4.3.1.2
4.3.1.23
4.3.1.24
4.3.1.25
1.4.1.
1.4.99.1
1.4.2.-
α-Amino acids (Nitrogenous compounds)
Nitrite
1.7.3.4
Hydroxylamine
Nitroalkane
1.13.12.16
1.7.3.1
1.13.12.-
1.7.1.10
1.7.99.1
L-Aspartate
6.3.1.1
6.3.1.4
L-Asparagine
4.3.1.1
3.5.1.38
3.5.1.1
L-Aspartate
Alanine, aspartate and glutamate metabolism
1.4.1.2
1.4.1.3
1.4.1.4
Nitrate
1.7.7.2
1.9.6.1
1.7.1.1
1.7.1.2
1.7.1.3
Nitrite
1.7.1.4
1.7.7.1
1.7.2.2
Ammonia
L-Glutamine
6.3.1.2
3.5.1.2
3.5.1.38
1.4.1.13
6.3.5.4
1.4.1.14
1.4.7.1
L-Glutamate
2.6.1.
1.7.99.4
1.18.6.1
1.19.6.1
1.7.2.1
1.4.3.
4.3.1.
1.14.13.35
1.14.12.1
4.3.1.
4.4.1.1
4.4.1.2
4.4.1.8
Nitric oxide
1.7.99.7
Dinitrogen oxide
1.7.99.6
Nitrogen
1.4.3.
Amines
3.5.1.
Amides
Cyclic amidines
3.5.4.
3.5.3.
Amidines
3.5.5.1
3.5.5.2
Nitriles
00910 10/14/10
(c) Kanehisa Laboratories

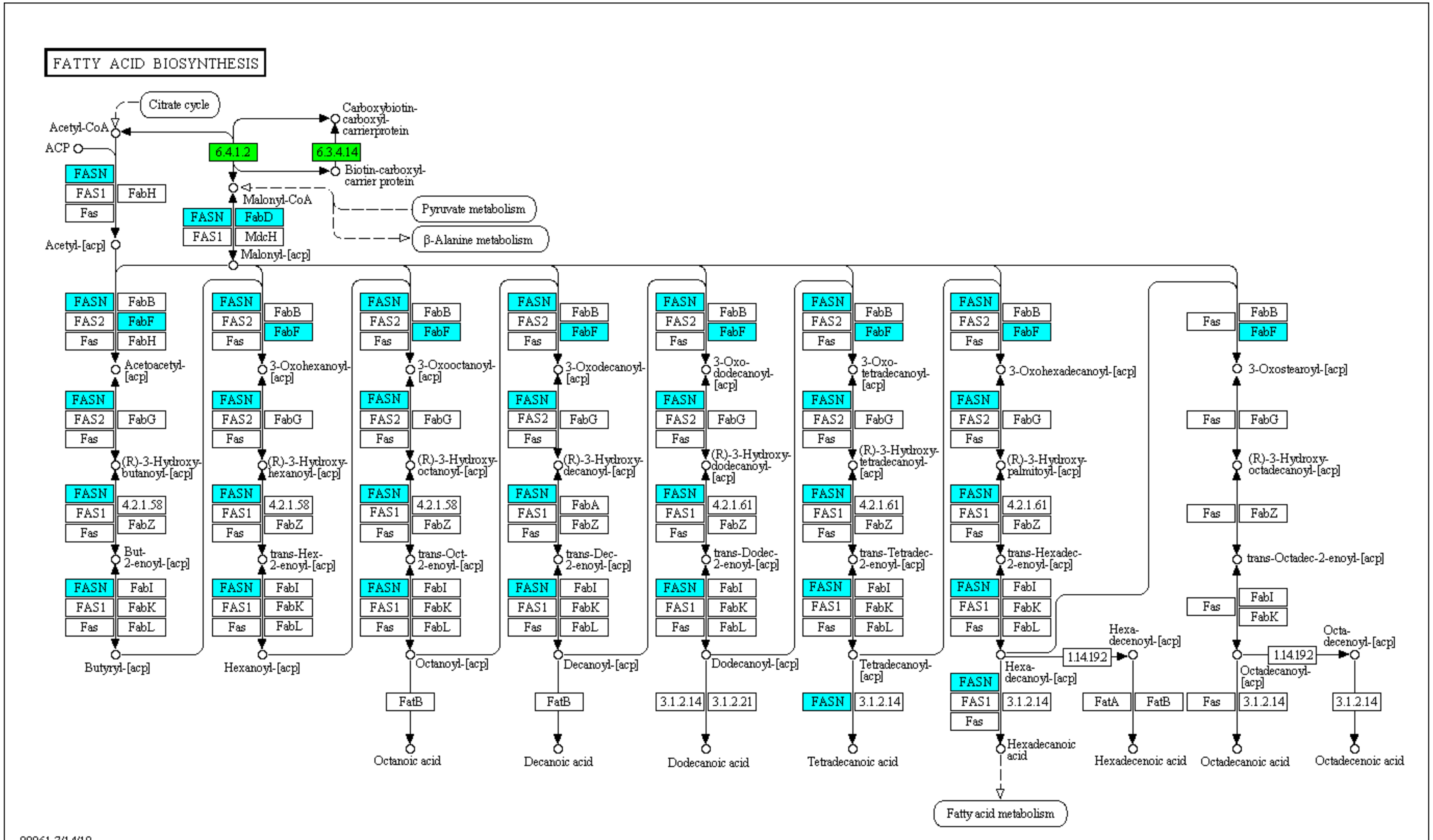

00061 7/14/10

(c) Kanehisa Laboratories

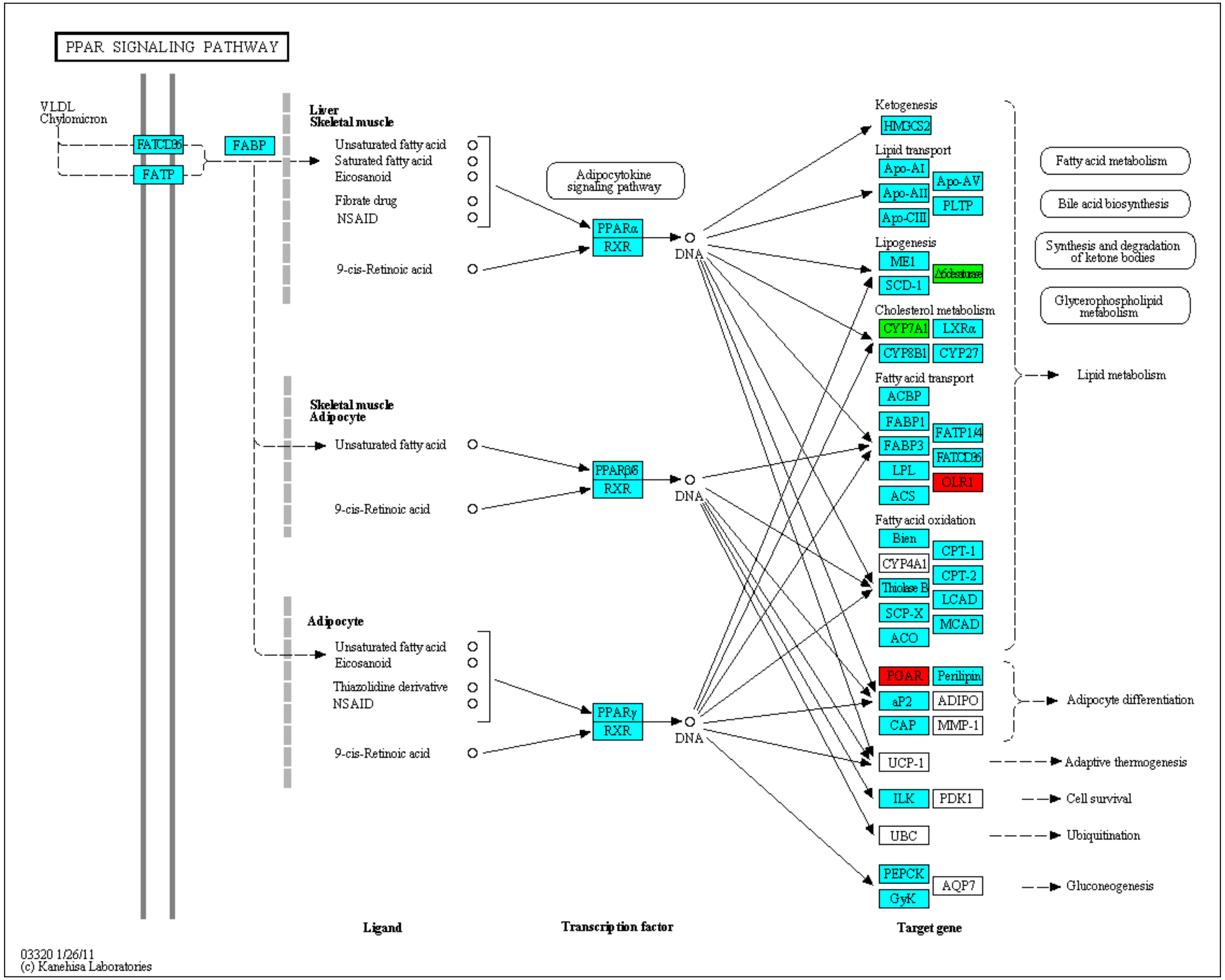
PPAR SIGNALING PATHWAY
VLDL
Chylomicron
FAT/CD36
FATP
FABP
Liver
Skeletal muscle
Unsaturated fatty acid
Saturated fatty acid
Eicosanoid
Fibrate drug
NSAID
9-cis-Retinoic acid
Adipocytokine signaling pathway
PPARα
RXR
DNA
Skeletal muscle
Adipocyte
Unsaturated fatty acid
9-cis-Retinoic acid
PPARβ/δ
RXR
DNA
Adipocyte
Unsaturated fatty acid
Eicosanoid
Thiazolidine derivative
NSAID
9-cis-Retinoic acid
PPARγ
RXR
DNA
Ketogenesis
HMGCS2
Lipid transport
Apo-AI
Apo-AII
Apo-CIII
Apo-AV
PLTP
Lipogenesis
ME1
SCD-1
Δ6desaturase
Cholesterol metabolism
CYP7A1
LXRα
CYP8B1
CYP27
Fatty acid transport
ACBP
FABP1
FABP3
LPL
ACS
FATP1/4
FAT/CD36
OLR1
Fatty acid oxidation
Bien
CYP4A1
Thiolase B
SCP-X
ACO
CPT-1
CPT-2
LCAD
MCAD
PGAR
Perilipin
aP2
ADIPO
CAP
MMP-1
UCP-1
ILK
PDK1
UBC
PEPCK
GyK
AQP7
Fatty acid metabolism
Bile acid biosynthesis
Synthesis and degradation of ketone bodies
Glycerophospholipid metabolism
Lipid metabolism
Adipocyte differentiation
Adaptive thermogenesis
Cell survival
Ubiquitination
Gluconeogenesis
Ligand
Transcription factor
Target gene
03320 1/26/11
(c) Kanehisa Laboratories

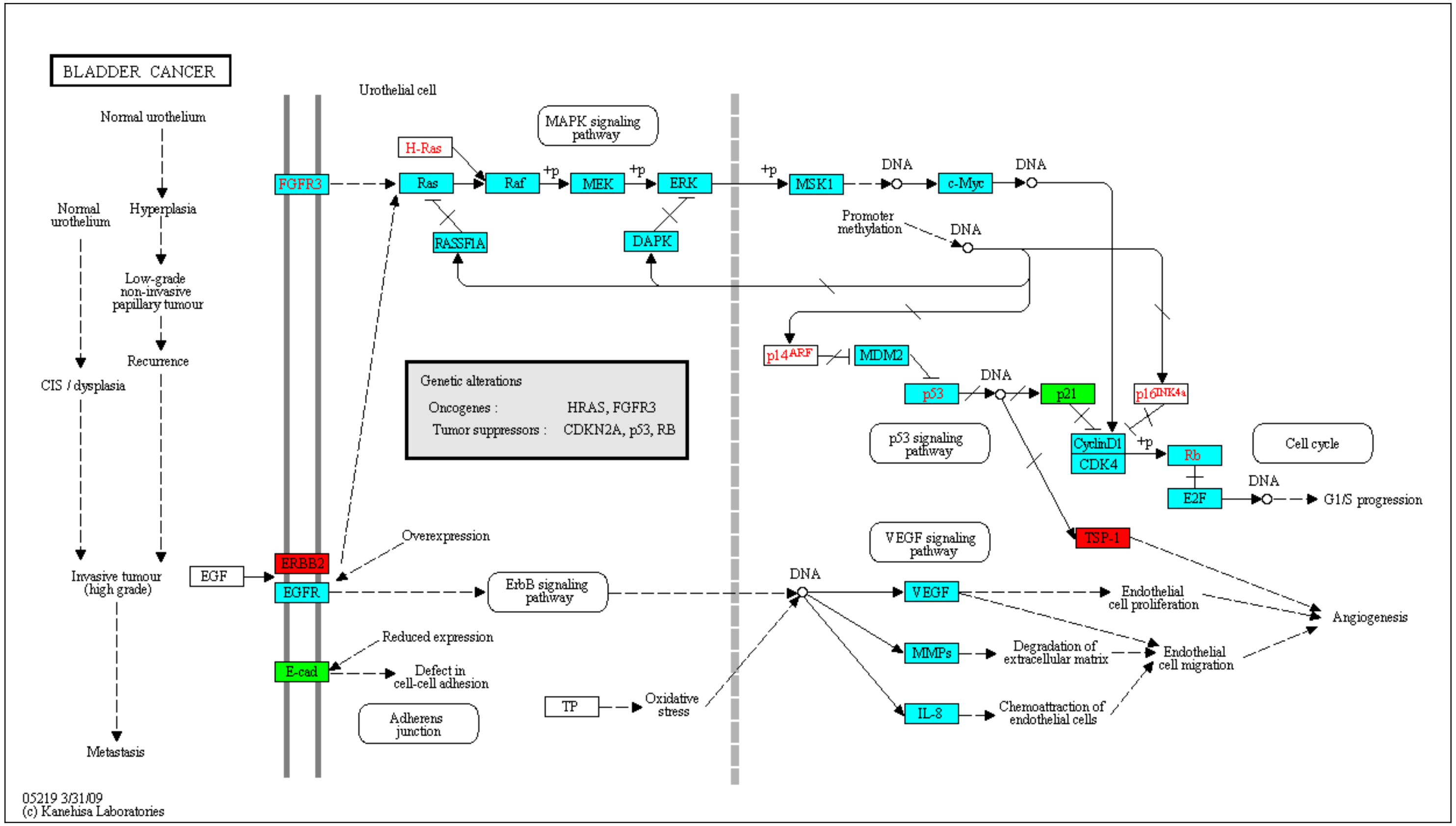
BLADDER CANCER
Normal urothelium
Hyperplasia
Normal urothelium
Low-grade non-invasive papillary tumour
Recurrence
CIS / dysplasia
Invasive tumour (high grade)
Metastasis
Urothelial cell
MAPK signaling pathway
H-Ras
FGFR3
Ras
Raf
MEK
ERK
+p
MSK1
DNA
c-Myc
RASSF1A
DAPK
Promoter methylation
p14ARF
MDM2
p53
p21
p16INK4a
CyclinD1
CDK4
Rb
E2F
G1/S progression
p53 signaling pathway
Cell cycle
Genetic alterations
Oncogenes : HRAS, FGFR3
Tumor suppressors : CDKN2A, p53, RB
Overexpression
ERBB2
EGF
EGFR
ErbB signaling pathway
VEGF signaling pathway
TSP-1
VEGF
Endothelial cell proliferation
Angiogenesis
MMPs
Degradation of extracellular matrix
Endothelial cell migration
IL-8
Chemoattraction of endothelial cells
Reduced expression
E-cad
Defect in cell-cell adhesion
Adherens junction
TP
Oxidative stress
05219 3/31/09
(c) Kanehisa Laboratories

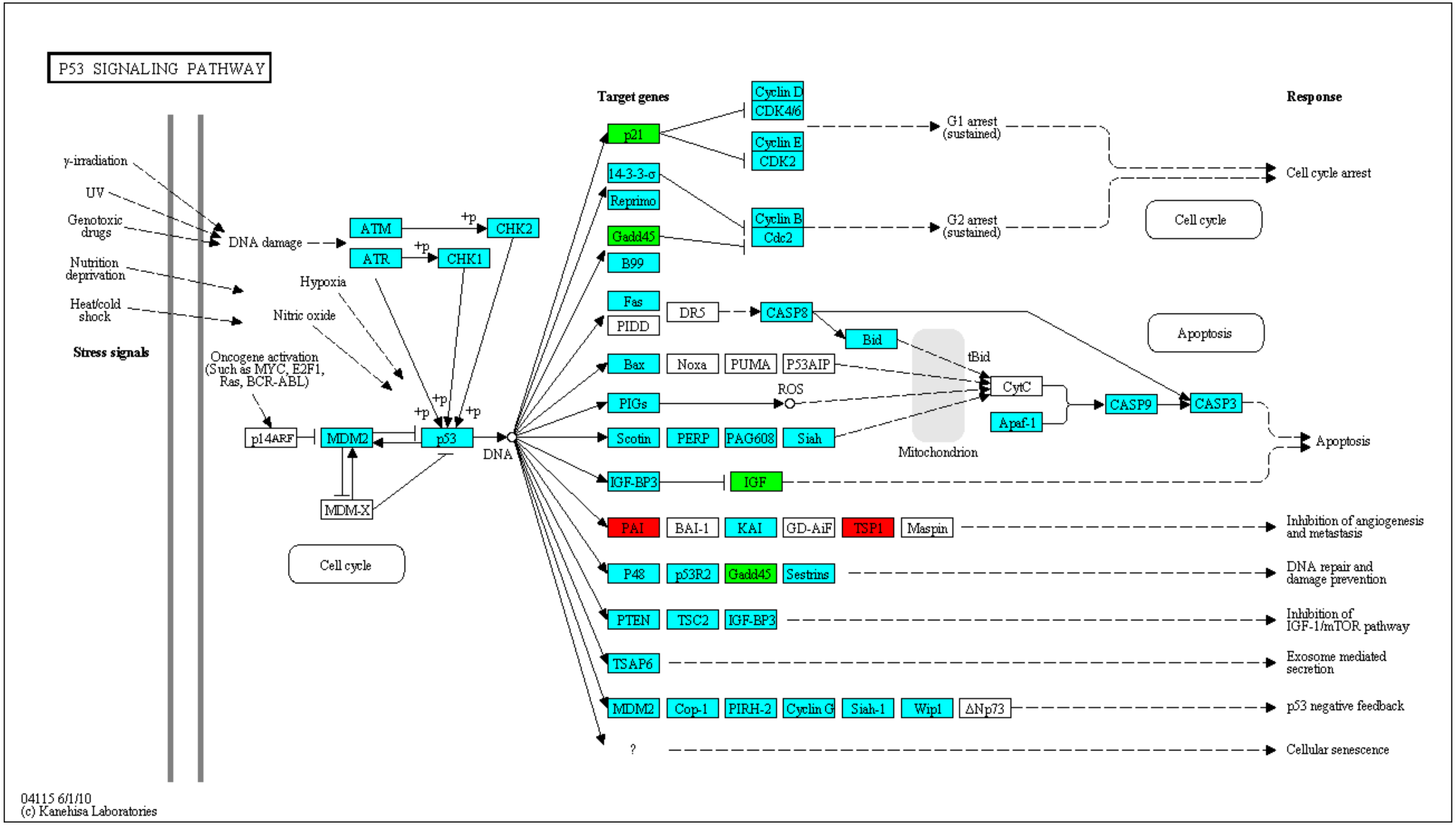

P53 SIGNALING PATHWAY
Target genes
Response
γ-irradiation
UV
Genotoxic drugs
Nutrition deprivation
Heat/cold shock
Stress signals
DNA damage
Hypoxia
Nitric oxide
Oncogene activation (Such as MYC, E2F1, Ras, BCR-ABL)
ATM
ATR
CHK2
CHK1
+p
p14ARF
MDM2
p53
DNA
MDM-X
Cell cycle
p21
14-3-3-σ
Reprimo
Gadd45
B99
Cyclin D
CDK4/6
Cyclin E
CDK2
Cyclin B
Cdc2
G1 arrest (sustained)
G2 arrest (sustained)
Cell cycle arrest
Cell cycle
Fas
PIDD
DR5
CASP8
Bid
tBid
Apoptosis
Bax
Noxa
PUMA
P53AIP
CytC
PIGs
ROS
Scotin
PERP
PAG608
Siah
Apaf-1
CASP9
CASP3
Mitochondrion
Apoptosis
IGF-BP3
IGF
PAI
BAI-1
KAI
GD-AiF
TSP1
Maspin
Inhibition of angiogenesis and metastasis
P48
p53R2
Gadd45
Sestrins
DNA repair and damage prevention
PTEN
TSC2
IGF-BP3
Inhibition of IGF-1/mTOR pathway
TSAP6
Exosome mediated secretion
MDM2
Cop-1
PIRH-2
Cyclin G
Siah-1
Wip1
ΔNp73
p53 negative feedback
?
Cellular senescence
04115 6/1/10
(c) Kanehisa Laboratories

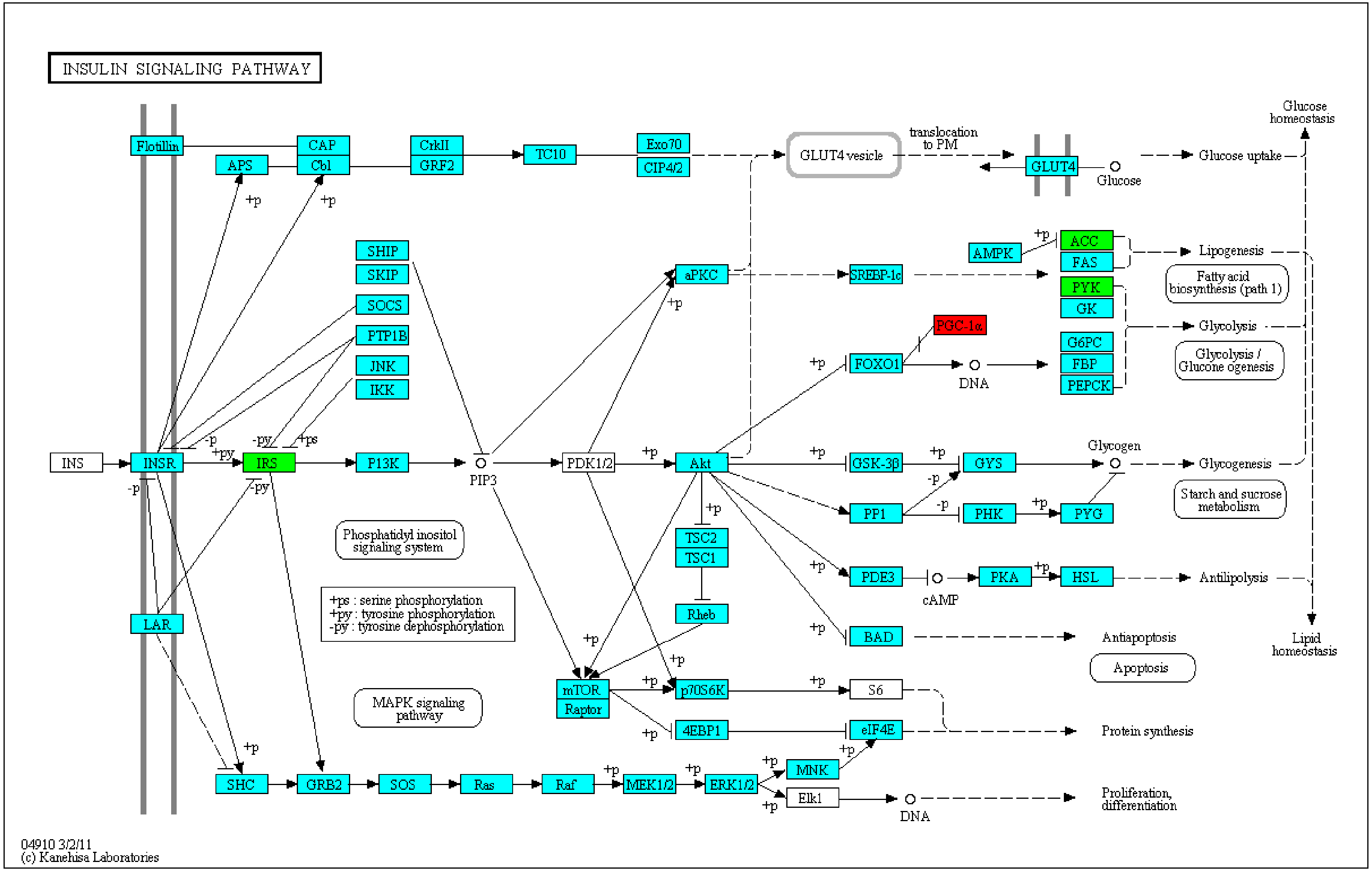
INSULIN SIGNALING PATHWAY
Flotillin
CAP
Cbl
APS
CrkII
GRF2
TC10
Exo70
CIP4/2
GLUT4 vesicle
translocation to PM
GLUT4
Glucose
Glucose uptake
Glucose homeostasis
+p
SHIP
SKIP
SOCS
PTP1B
JNK
IKK
aPKC
SREBP-1c
AMPK
ACC
FAS
Lipogenesis
Fatty acid biosynthesis (path 1)
PYK
GK
Glycolysis
PGC-1α
FOXO1
DNA
G6PC
FBP
PEPCK
Glycolysis / Glucone ogenesis
INS
INSR
IRS
PI3K
PIP3
PDK1/2
Akt
GSK-3β
GYS
Glycogen
Glycogenesis
-p
+py
-py
+ps
PP1
PHK
PYG
Starch and sucrose metabolism
TSC2
TSC1
Rheb
PDE3
cAMP
PKA
HSL
Antilipolysis
Lipid homeostasis
BAD
Antiapoptosis
Apoptosis
Phosphatidyl inositol signaling system
+ps : serine phosphorylation
+py : tyrosine phosphorylation
-py : tyrosine dephosphorylation
LAR
mTOR
Raptor
p70S6K
S6
4EBP1
eIF4E
Protein synthesis
MAPK signaling pathway
SHC
GRB2
SOS
Ras
Raf
MEK1/2
ERK1/2
MNK
Elk1
Proliferation, differentiation
04910 3/2/11
(c) Kanehisa Laboratories

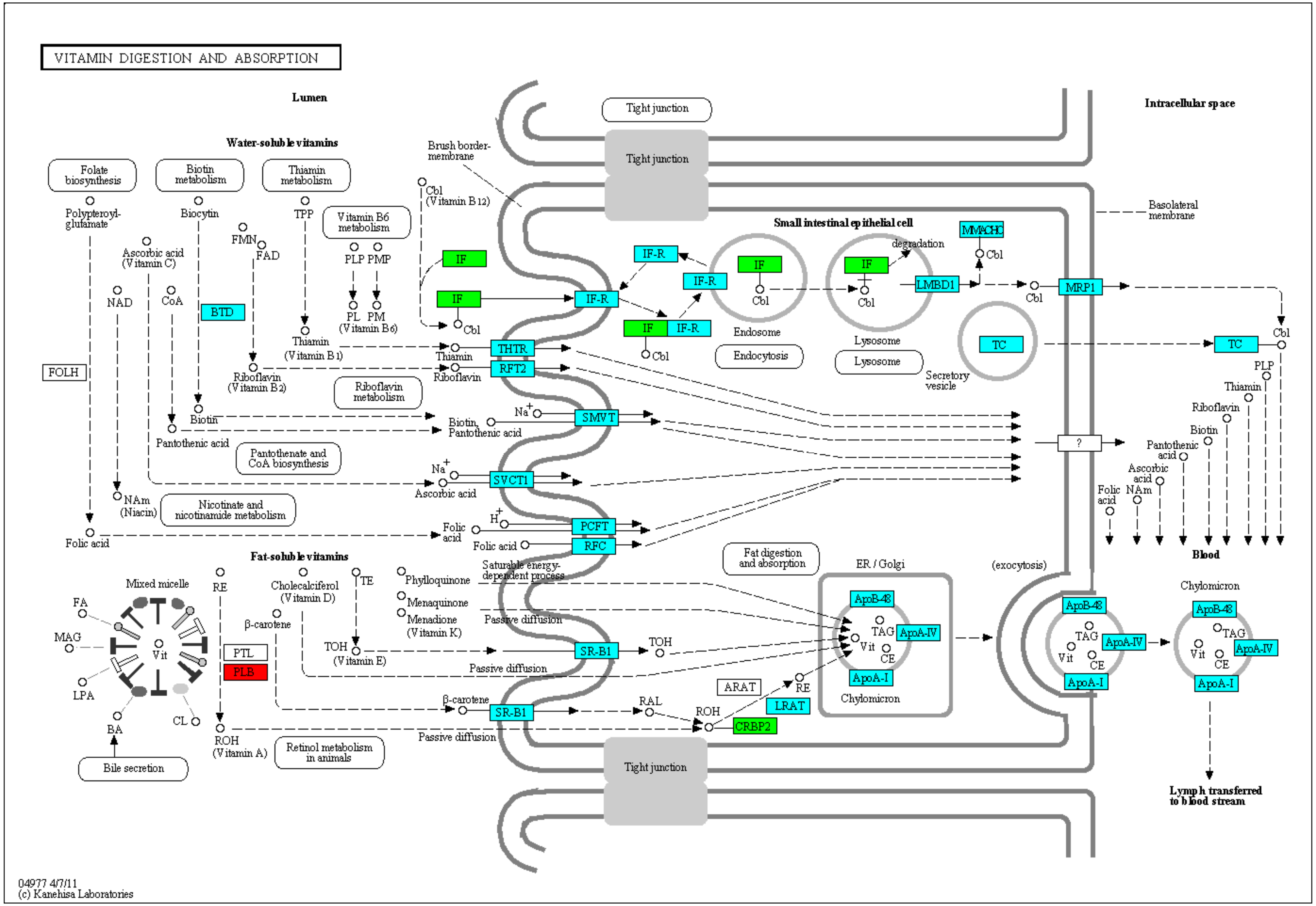
VITAMIN DIGESTION AND ABSORPTION
Lumen
Intracellular space
Water-soluble vitamins
Folate biosynthesis
Biotin metabolism
Thiamin metabolism
Vitamin B6 metabolism
Polypteroyl-glutamate
Biocytin
TPP
Brush border-membrane
Cbl (Vitamin B12)
Tight junction
Basolateral membrane
Small intestinal epithelial cell
MMACHC
degradation
Ascorbic acid (Vitamin C)
FMN
FAD
PLP PMP
PL PM (Vitamin B6)
NAD
CoA
BTD
IF
IF-R
LMBD1
MRP1
Cbl
Endosome
Endocytosis
Lysosome
TC
Secretory vesicle
FOLH
Thiamin (Vitamin B1)
Riboflavin (Vitamin B2)
Riboflavin metabolism
THTR
RFT2
Thiamin
Riboflavin
PLP
Biotin
Pantothenic acid
Pantothenate and CoA biosynthesis
Na^{+}
SMVT
Biotin, Pantothenic acid
SVCT1
Ascorbic acid
?
NAm (Niacin)
Nicotinate and nicotinamide metabolism
H^{+}
PCFT
RFC
Folic acid
Folic acid NAm
Blood
Fat-soluble vitamins
Fat digestion and absorption
ER / Golgi
(exocytosis)
Chylomicron
Mixed micelle
RE
Cholecalciferol (Vitamin D)
TE
Phylloquinone
Saturable energy-dependent process
Menaquinone
Menadione (Vitamin K)
Passive diffusion
FA
MAG
Vit
LPA
CL
BA
Bile secretion
β-carotene
PTL
PLB
TOH (Vitamin E)
SR-B1
TOH
ApoB-48
TAG
ApoA-IV
CE
ApoA-I
ARAT
LRAT
RAL
ROH
CRBP2
ROH (Vitamin A)
Retinol metabolism in animals
Lymph transferred to blood stream
04977 4/7/11
(c) Kanehisa Laboratories

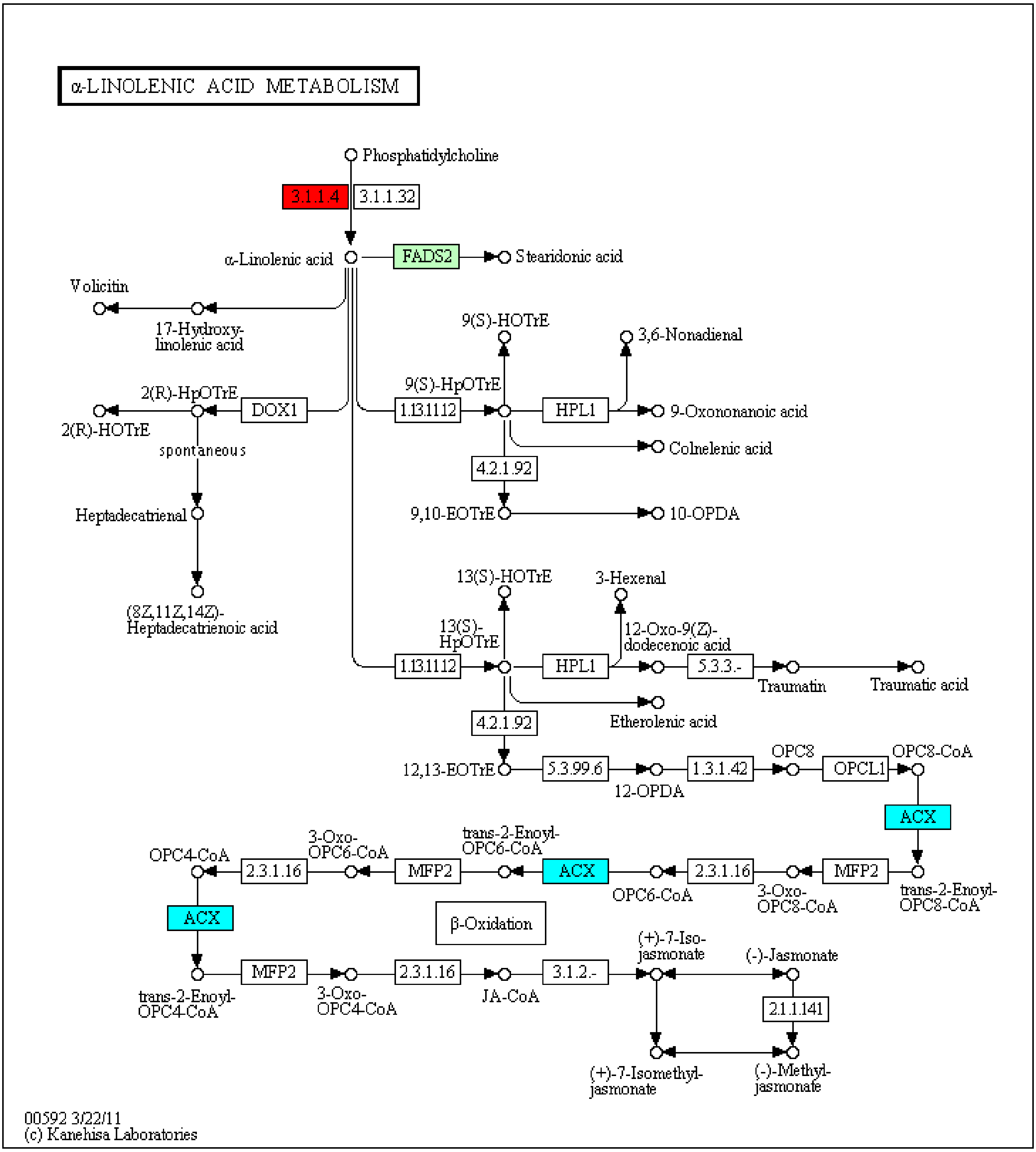
α-LINOLENIC ACID METABOLISM
Phosphatidylcholine
3.1.1.4
3.1.1.32
α-Linolenic acid
FADS2
Stearidonic acid
Volicitin
17-Hydroxy-linolenic acid
9(S)-HOTrE
3,6-Nonadienal
2(R)-HpOTrE
DOX1
9(S)-HpOTrE
1.13.11.12
HPL1
9-Oxononanoic acid
2(R)-HOTrE
spontaneous
Colnelenic acid
4.2.1.92
Heptadecatrienal
9,10-EOTrE
10-OPDA
13(S)-HOTrE
3-Hexenal
(8Z,11Z,14Z)-Heptadecatrienoic acid
13(S)-HpOTrE
12-Oxo-9(Z)-dodecenoic acid
1.13.11.12
HPL1
5.3.3.-
Traumatin
Traumatic acid
Etherolenic acid
4.2.1.92
OPC8
OPC8-CoA
12,13-EOTrE
5.3.99.6
1.3.1.42
OPCL1
12-OPDA
ACX
3-Oxo-OPC6-CoA
trans-2-Enoyl-OPC6-CoA
OPC4-CoA
2.3.1.16
MFP2
ACX
2.3.1.16
MFP2
OPC6-CoA
3-Oxo-OPC8-CoA
trans-2-Enoyl-OPC8-CoA
ACX
β-Oxidation
(+)-7-Iso-jasmonate
(-)-Jasmonate
MFP2
2.3.1.16
3.1.2.-
trans-2-Enoyl-OPC4-CoA
3-Oxo-OPC4-CoA
JA-CoA
2.1.1.141
(+)-7-Isomethyl-jasmonate
(-)-Methyl-jasmonate
00592 3/22/11
(c) Kanehisa Laboratories

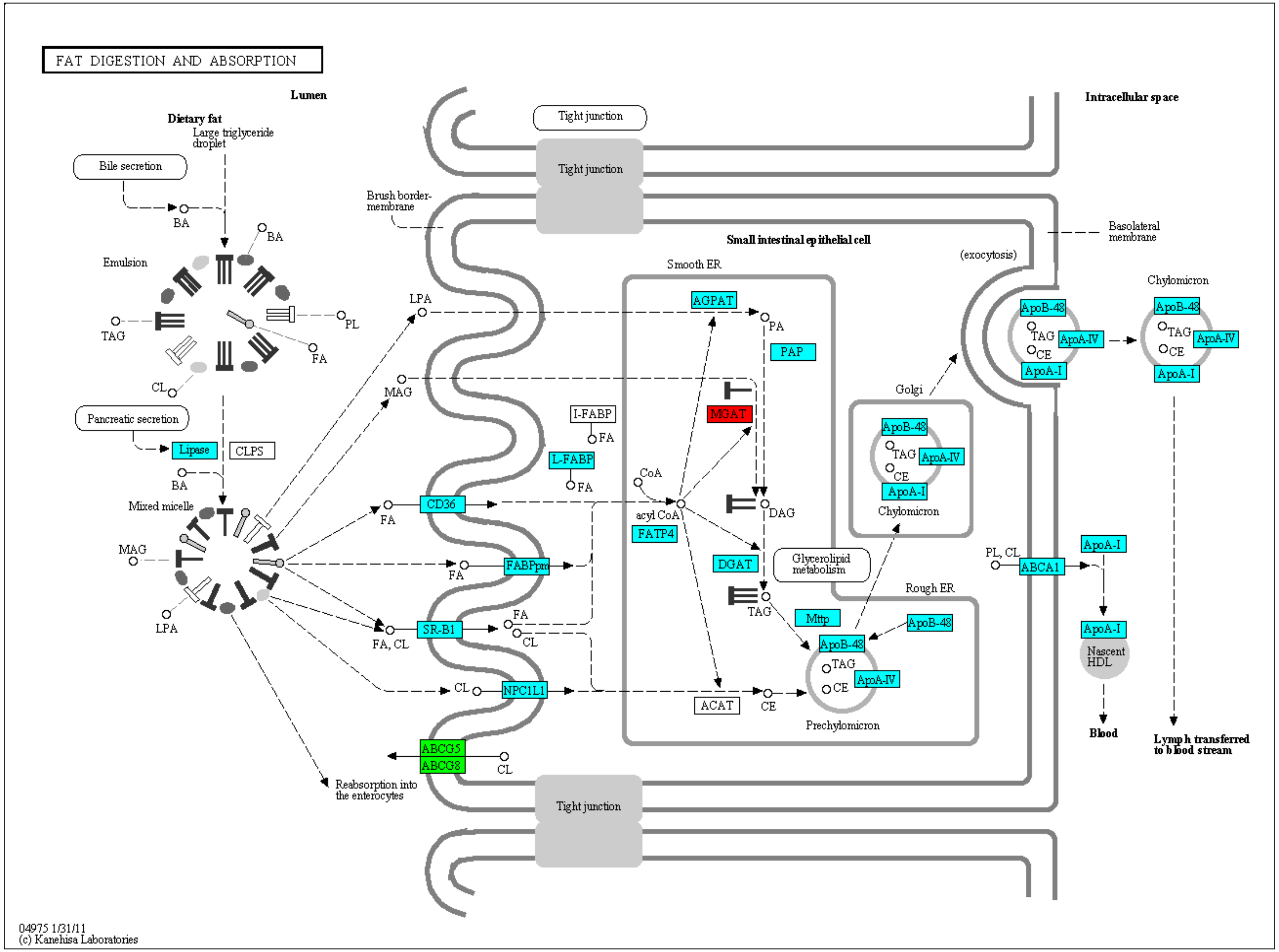

FAT DIGESTION AND ABSORPTION
Lumen
Dietary fat
Large triglyceride droplet
Bile secretion
BA
Emulsion
TAG
PL
FA
CL
Pancreatic secretion
Lipase
CLPS
Mixed micelle
MAG
LPA
Brush border-membrane
Tight junction
Small intestinal epithelial cell
Smooth ER
AGPAT
PA
PAP
MGAT
DAG
I-FABP
L-FABP
CoA
acyl CoA
FATP4
DGAT
Glycerolipid metabolism
TAG
Mttp
ApoB-48
ApoA-IV
CE
Prechylomicron
Rough ER
ACAT
CD36
FABPpm
SR-B1
FA, CL
NPC1L1
ABCG5
ABCG8
Reabsorption into the enterocytes
Golgi
ApoA-I
Chylomicron
(exocytosis)
Basolateral membrane
Intracellular space
PL, CL
ABCA1
Nascent HDL
Blood
Lymph transferred to blood stream
04975 1/31/11
(c) Kanehisa Laboratories

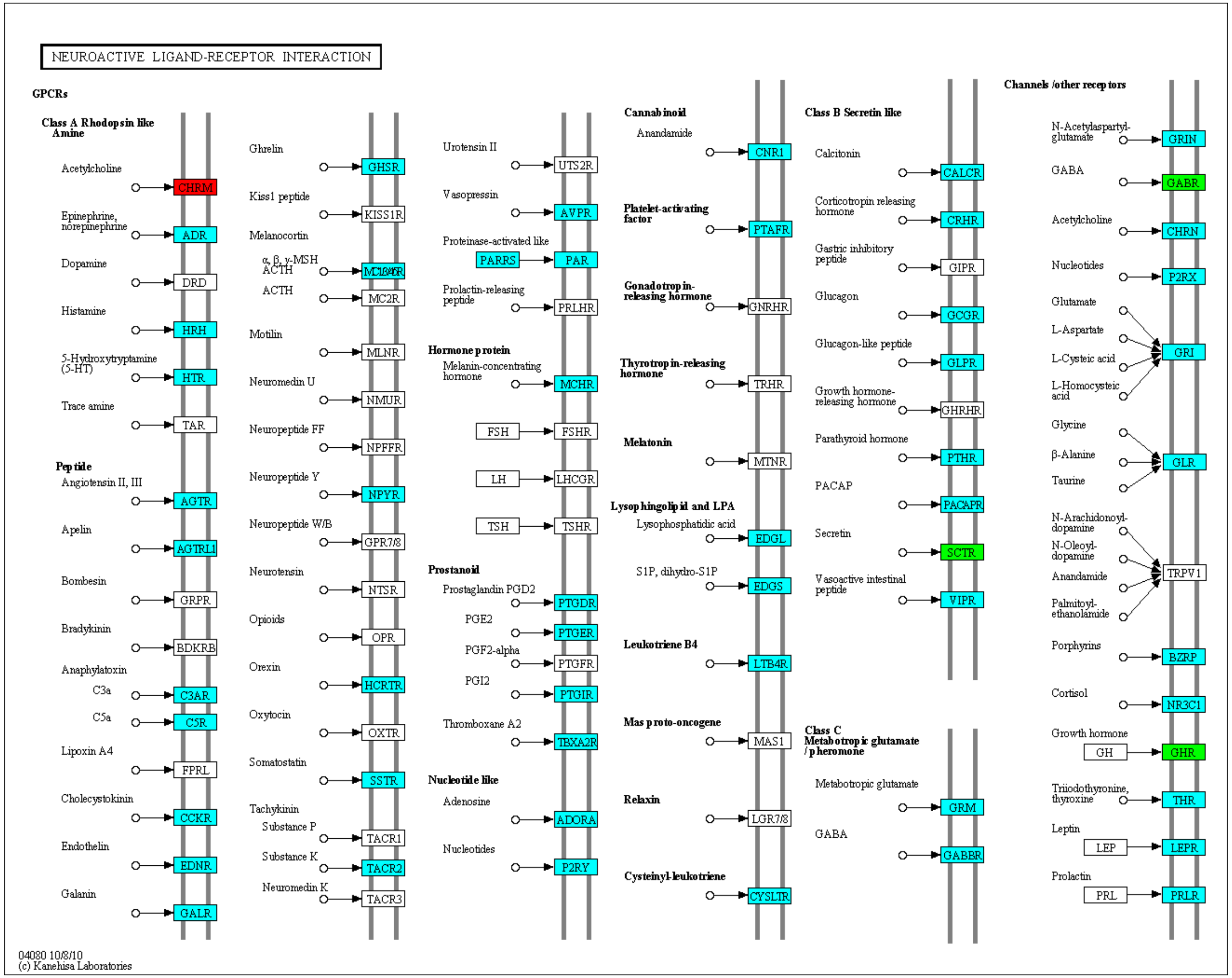

NEUROACTIVE LIGAND-RECEPTOR INTERACTION
GPCRs
Class A Rhodopsin like
Amine
Acetylcholine
CHRM
Epinephrine, norepinephrine
ADR
Dopamine
DRD
Histamine
HRH
5-Hydroxytryptamine (5-HT)
HTR
Trace amine
TAR
Peptide
Angiotensin II, III
AGTR
Apelin
AGTRL1
Bombesin
GRPR
Bradykinin
BDKRB
Anaphylatoxin
C3a
C3AR
C5a
C5R
Lipoxin A4
FPRL
Cholecystokinin
CCKR
Endothelin
EDNR
Galanin
GALR
Ghrelin
GHSR
Kiss1 peptide
KISS1R
Melanocortin
α, β, γ-MSH
ACTH
MC1/3/4/5R
ACTH
MC2R
Motilin
MLNR
Neuromedin U
NMUR
Neuropeptide FF
NPFFR
Neuropeptide Y
NPYR
Neuropeptide W/B
GPR7/8
Neurotensin
NTSR
Opioids
OPR
Orexin
HCRTR
Oxytocin
OXTR
Somatostatin
SSTR
Tachykinin
Substance P
TACR1
Substance K
TACR2
Neuromedin K
TACR3
Urotensin II
UTS2R
Vasopressin
AVPR
Proteinase-activated like
PARRS
PAR
Prolactin-releasing peptide
PRLHR
Hormone protein
Melanin-concentrating hormone
MCHR
FSH
FSHR
LH
LHCGR
TSH
TSHR
Prostanoid
Prostaglandin PGD2
PTGDR
PGE2
PTGER
PGF2-alpha
PTGFR
PGI2
PTGIR
Thromboxane A2
TBXA2R
Nucleotide like
Adenosine
ADORA
Nucleotides
P2RY
Cannabinoid
Anandamide
CNR1
Platelet-activating factor
PTAFR
Gonadotropin-releasing hormone
GNRHR
Thyrotropin-releasing hormone
TRHR
Melatonin
MTNR
Lysophingolipid and LPA
Lysophosphatidic acid
EDGL
S1P, dihydro-S1P
EDGS
Leukotriene B4
LTB4R
Mas proto-oncogene
MAS1
Relaxin
LGR7/8
Cysteinyl-leukotriene
CYSLTR
Class B Secretin like
Calcitonin
CALCR
Corticotropin releasing hormone
CRHR
Gastric inhibitory peptide
GIPR
Glucagon
GCGR
Glucagon-like peptide
GLPR
Growth hormone-releasing hormone
GHRHR
Parathyroid hormone
PTHR
PACAP
PACAPR
Secretin
SCTR
Vasoactive intestinal peptide
VIPR
Class C
Metabotropic glutamate /pheromone
Metabotropic glutamate
GRM
GABA
GABBR
Channels /other receptors
N-Acetylaspartyl-glutamate
GRIN
GABA
GABR
Acetylcholine
CHRN
Nucleotides
P2RX
Glutamate
L-Aspartate
L-Cysteic acid
L-Homocysteic acid
GRI
Glycine
β-Alanine
Taurine
GLR
N-Arachidonoyl-dopamine
N-Oleoyl-dopamine
Anandamide
Palmitoyl-ethanolamide
TRPV1
Porphyrins
BZRP
Cortisol
NR3C1
Growth hormone
GH
GHR
Triiodothyronine, thyroxine
THR
Leptin
LEP
LEPR
Prolactin
PRL
PRLR
04080 10/8/10
(c) Kanehisa Laboratories
