# Supplementary material for: RNA-seq analysis of differential gene expression in liver from lactating dairy cows divergent in negative energy balance
Source: BMC Genomics. 2012 May 20;13:193. doi: 10.1186/1471-2164-13-193 (PMC3465249; doi:10.1186/1471-2164-13-193)
Supplement: Additional file 6 — Maps of KEGG pathways associated with all SDE (FDR 0.1%) genes. [file 1471-2164-13-193-S6.doc]

**
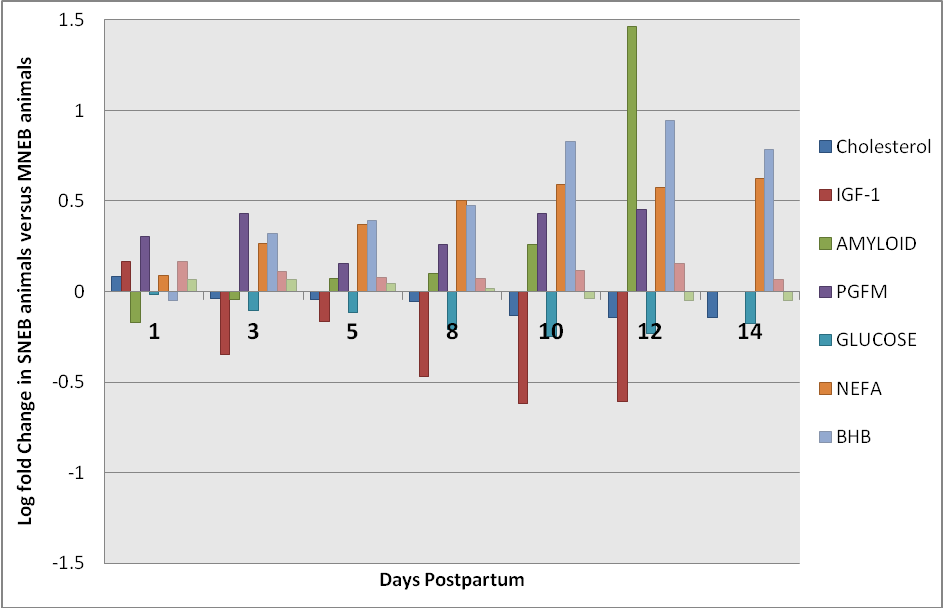
**

**Additional file 6** Effect of treatment on various blood metabolites in the SNEB versus MNEB animals.
